# Self-Confirmation and Ascertainment of the Candidate Genomic Regions of Complex Trait Loci – A None-Experimental Solution

**DOI:** 10.1371/journal.pone.0153676

**Published:** 2016-05-20

**Authors:** Lishi Wang, Yan Jiao, Yongjun Wang, Mengchen Zhang, Weikuan Gu

**Affiliations:** 1 Department of Orthopedic Surgery & BME, -Campbell-Clinic, University of Tennessee Health Science Center, Memphis, TN, 38163, United States of America; 2 Mudanjiang Medical College, Mudanjiang, Heilongjiang, 157001, PR China; 3 Department. of Neurology, Beijing Tiantan Hospital, Capital Medical University, Beijing, 100050, PR China; 4 National Center of Soybean Research, Institute of Hebei Cereal and Oil Crops, Hebei Academy of Agriculture and Forestry Sciences, Shijiazhuang, Hebei, 050011, PR China; 5 Research Service, Veterans Affairs Medical Center, 1030 Jefferson Avenue, Memphis, TN, 38104, United States of America; 6 Department of Basic Research, Inner Mongolia Medical University, Inner Mongolia, 010110, PR China; Mahatma Phule Agricultural University, INDIA

## Abstract

Over the past half century, thousands of quantitative trait loci (QTL) have been identified by using animal models and plant populations. However, the none-reliability and imprecision of the genomic regions of these loci have remained the major hurdle for the identification of the causal genes for the correspondent traits. We used a none-experimental strategy of strain number reduction for testing accuracy and ascertainment of the candidate region for QTL. We tested the strategy in over 400 analyses with data from 47 studies. These studies include: 1) studies with recombinant inbred (RI) strains of mice. We first tested two previously mapped QTL with well-defined genomic regions; We then tested additional four studies with known QTL regions; and finally we examined the reliability of QTL in 38 sets of data which are produced from relatively large numbers of RI strains, derived from C57BL/6J (B6) X DBA/2J (D2), known as BXD RI mouse strains; 2) studies with RI strains of rats and plants; and 3) studies using F2 populations in mice, rats and plants. In these cases, our method identified the reliability of mapped QTL and localized the candidate genes into the defined genomic regions. Our data also suggests that LRS score produced by permutation tests does not necessarily confirm the reliability of the QTL. Number of strains are not the reliable indicators for the accuracy of QTL either. Our strategy determines the reliability and accuracy of the genomic region of a QTL without any additional experimental study such as congenic breeding.

## Introduction

Thousands of quantitative trait loci (QTL) influencing complex traits have been genetically mapped in the last half century. However, the identification of gene(s) comprising the QTL (QTGs) has remained a difficulty in the study of complex traits. The common challenges for identification of QTG are the unreliability of the locus and poorly defined or ambiguous boundaries of the QTL. Recently, high-throughput technologies and whole genome SNPs have provided dense markers for mapping. However, quality of genotyping and phenotypic data and other experimental errors remain the issue in the determination of reliability and accuracy of the QTL. For most studies, ascertainment of QTL has mainly relied on the confirmation from different studies or the congenic breeding, which require extensive resources and are time consuming. In many cases, different QTL for the same trait from different studies have caused confusion [1]. Thus, regardless how condensed and accurate the genomic markers are, the uncertainty of the QTL locations prevents the utilization of the data for the further study and clinic applications, if any. Resolution of this long historic problem would open the door to characterize and understand the functional pathways underlying complex traits and ultimately implement treatments for complex disorders. Our investigation in this study focuses on the determinant of accuracy of the mapping and the reliability of the genomic region of the candidate genes.

## Materials and Methods

### Data sets used for the study of RI strains

This study utilizes the data sets that were collected by multiple investigators. Two sets of data were first used to determine the minimum percentage of strain reduction to ensure the reliability of the QTL. The first set of data contains relatively large number of RI strains in which the locations of the QTL have been confirmed in multiple tests. This set of data included two piece of data. The first one is data of bone cross section of tibiae, GeneNetwork (ID 13054 of 46 BXD RI strains with two parental strains for the repeatability of QTL with sequential reduction of number of strains. The second piece is the set of data from a pair of data for cerebral cortex volumes bilaterally in 54 BXD RI (ID 10997) with shrinkage corrected only and data adjusted for shrinkage, age, sex, plane of section [2].

The second set of data includes BMD data from early studies of BMD with less number of strains. These early studies include a study in BMD corrected for whole body weight of both sexes [3] in 2001 (GeneNetwork ID 10443), and bone cross-sectional area of both sexes in 2002 [4] (GeneNetwork ID 10360).

Additional four sets of data were used for confirmation of the application of our strategy in RI strains, F2 populations, and in mice, rats and plants.

The first set of data is additional four analyses using mouse RI strain with ID12569, ID 12567, ID 12971, and ID10866 [5–8].

The second set of data includes 38 pieces of data for a variety of phenotypes to confirm the reliability of QTL based on the results from the first and second sets of strains. *These data include*: ID 11476 with data from 63 RI strains, ID 11730 with data from 64 RIstrains, ID 10865 with data from 69 RI strains, ID 10891 with data from 58 RI strains, ID11023 with data from 78 RI strains, 11024 with data from 56 RI strains, ID 11033 with data from 57 RI strains, ID12297 with data from 66 strains, ID12344 with data from 67 RI strains, ID12436 with data from 66 RI strains, ID12463 with data from 68 RI strains, ID 12485 with data from 44 RI strains, ID 12568 with data from 43 RI strains, ID12577 with data from 45 RI strains, ID12659 with data from 88 RI strains, ID12667 with data from 38 RI strains, ID12668 with data from 38 RI strains, ID12683 with data from 61 RI strains, ID12685 with data from 88 RI strains, ID12911 with data from 42 RI strains, ID12981 with data from 42 RI strains, ID12983 with data from 42 RI strains, ID13548 with data from 55 RI strains, ID13550 with data from 56 RI strains, ID13551 with data from 68 RI strains, ID13587 with data from 70 RI strains, ID14781 with data from 68 RI strains, ID16180 with data from 61RI strains, ID16185 with data from 35 RI strains, ID11960 with data from 62 RI strains, ID11983 with data from 64 RI strains, ID12009 with data from 63 RI strains, ID12012 with data from 63 RI strains, ID11963 with data from 62 RI strains, ID12472 with data from 63 RI strains, ID11031 with data from 29 RI strains, ID12546 with data from 24 RI strains, and ID11029 with data from 29 RI strains.

The third set of data is from rat RI strains. Currently, the RI strains of rats at GeneNetwork were derived from a cross between the spontaneously hypertensive rat (SHR/OlaIpcv = H) and Brown Norway (BN.Lx/Cub or BN = B). The HXB/BXH Genotype Database was assembled by RW Williams and Michal Pravenec and colleagues using a compendium of approximately 1100 markers that have been typed over the past decade. We used two sets of RI strains of rats for testing the ability of method of sequential reduction to determine the reliability of the QTL. The first set of data is for the phenotype of body weight of mothers (ID 10101). The second set of data is for the alcohol response of males evaluated by the two-bottle choice test (ID 10190).

The fourth set of data is from plants, which includes the data from Barley SMP Published Phenotypes Database ID 49919 and Soybean ID 5 Node number on main branch.

### Data sets used for the study of F2 population

Test of strategy of strain number reduction was conducted with F2 populations from animals and plants. The mouse F2 population is the B6BTBRF2 cross which consists of a subset of 60 F2 progeny generated by crossing C57BL/6J and BTBR strains. All of these cases are homozygous for the spontaneous obese mutation in the leptin gene (Lep-ob/ob). Liver gene expression data were generated by Hong Lan and Alan Attie at The University of Wisconsin-Madison. From this F2 population, we used two sets of data, one is in mice for Delta Ct numbers of phosphoenopyruvate carboxykinase (NM 011044) (ID 10017) measured by quantitative real-time RT-PCR. The other set of study is the body weight at 8 weeks of age (ID 10003).

Test also was conducted with a F2 population in rat model. We use the data of gene expression level of eye mRNA (Dataset ID 73, Name: UIOWA Eye mRNA RAE230v2). We tested the QTLs for growth factor, beta 1*(Tgfβ1)* expression level and for procollagen, type III, alpha 1 (Col3a1) expression level.

The plant population is a soybean F2 population generated by crossing J12XJ58F2. The population was created by Professor Mengchen Zhang at Institute of Hebei Cereal and Oil Crops, Hebei Academy of Agriculture and Forestry Sciences. The population data are from a total of 83 of individuals.

### Method of sequential reduction of strain number

**Fig 1:**
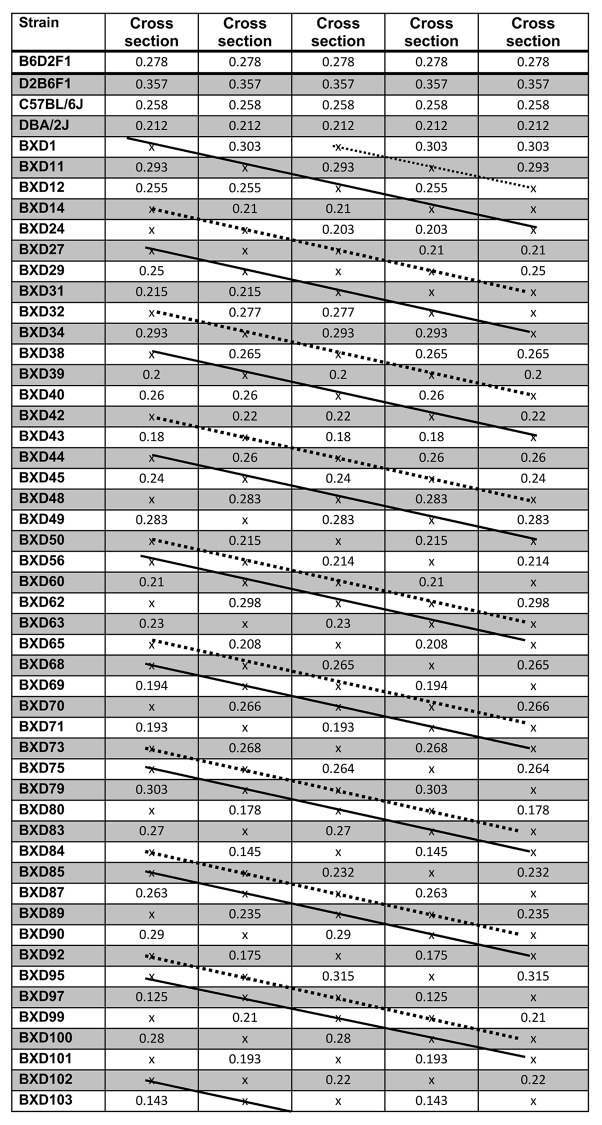
Methods of strain number reduction. Left column, name of the strains, top column, traits of the strains. The figure shows how the number of strains was reduced. The strains were reduced sequentially one by one in each test step. However, the individual strains eliminated in each step are done randomly. The sequencing reduction was first conducted using the data of bone cross section in five replicates. Solid line indicate the strains that was taken out in the first cycle of the tests. Dashed lines indicated the strains that were eliminated in the test in the second cycle of the tests.

The method we used is to re-map the QTL with reduced numbers of strains. The strain number reduction is done sequentially one by one in each test step (thus, 1, 2, 3, 4, ……) (Fig 1). However, the individual strains eliminated in each step are done randomly. The sequencing reduction was first conducted using the data of bone cross section. For each number reduction, we have five replicates. The number of reduction of samples starts with 1 till 36, thus the maximum reduction in the number of strains equals to 20%. For each reduction, a different strain was sequentially eliminated from the testing strains. For example, for the test of five replicates with 45 strains, the eliminated strains from each of the five testers are BXD1, BXD11, BXD12, BXD14 and BXD24; for the test with 44 strains, the eliminated two strains from each of the five testers are BXD1 and BXD27; BXD11 and BXD29; BXD12 and BXD31; BXD14 and BXD32; and BXD24 and BXD34. For the test with 43 strains, the eliminated three strains from each of the five testers are BXD1, BXD27 and BXD38; BXD11, BXD29 and BXD39; BXD12, BXD31 and BXD40; BXD14, BXD32 and BXD42; and BXD24, BXD34 and BXD43. As shown in Fig 1, the strain reduction in those tests forms a uniformed pattern, so that the reduction in those testing populations is equally performed.

### Testing strategy of strain number reduction

For the other sets of data, the strain number of reduction was conducted from 10% to 15% directly. The tests for RI strains in mice, rats, and plants are conducted with 5 replicates, each with 15% of strain number reduction. For the testing of F2 populations in mice, rats and plants, 10 replicates with reduced number of strains were used. For the testing of potential application in GWAS, 10 replicates with 15% reduction in size were used.

For these 400 analyses, we tested with 2000 permutations; and for the initial mapping of data of 47 studies, we conducted 2000 permutations and 2000 bootstrap tests.

### Determining whether the peak region of the QTL can be reliably located at the same genomic region with sequentially reduced number of strains

The procedure for identification of the peak genomic region of QTL is as the flowing: we first narrowed down the peak region by re-mapping the QTL until the top of QTL region appears flat at the Genenetwork. Next, we compare the locations of peak regions of the same QTL from all five replicates with reduced numbers of strains. We then retrieve the genotype information of all replicates that flank and within the plateaued QTL region. We finally compare the SNP blocks of the RI strains and identify the genomic at the peak region of the QTL.

After we ensured the genomic region at the peak region, we identify all the genes and other genetic components within the block using information of Ensembl. The candidate genes for some QTL in this study have been previously reported. For the confirmation of the accuracy of the method of strain number reduction, we then examine whether the previously identified QTG is within the list of genes within the peak genomic region.

For the tests with samples from rats and plants, we only examined the location of peak region of the QTL. Because the genomic sequencing and gene lists are not listed on the database of GeneNetwork, we were not able to identify the genes within the defined genomic region.

## Results

### Test of accuracy of QTL position mapped with large and small number of strains by sequential reduction of number of strains

Our first test is to use the strategy of sequential reduction number of strains to test the accuracy of the QTL mapping from studies using mouse recombinant inbred (RI) strains. Because of the existing of multiple data collected by different time or investigators for the same phenotypes in the GeneNetwork (http://www.genenetwork.org/webqtl/main.py), we were able to collect two sets of data to test the repeatability of mapping data.

At the very beginning, we conducted successions of analysis with data of cross section of tibiae of 46 BXD RI strains in the male mice (GeneNetwork ID 13054). With 2000 permutation tests, LRS criteria for the mapping with the original data of 46 strains are: suggestive level = 10.53, significant = 17.42, and highly significant = 20.34. Three QTL loci were detected (Figure A in S1 Text).

The major QTL is detected from the chromosome (Chr) 15, which has a LRS score of 22.295. We then tested the reproducibility by sequentially reducing the number of strains. For each set of test, we eliminated the data from one different strain in each of the five replicates, called biological replicates. Thus, in the first test, we have five sets of data, each contains 45 strains with one strain eliminated. We used those sets of data for the QTL mapping at GeneNetwork, with 2000 permutation tests in each mapping process. Next, we eliminated one additional different strain from each of those five sets of biological replicates. Then, we used those five sets of data each with 44 strains for the QTL analysis again. We continued the analysis cycle till the data from RI strains was reduced to 36 in each replicate (Table A in S1 Text). Thus, data from more than 20% of strains were eliminated from each of five sets of data at the last analytic cycle.

**Fig 2:**
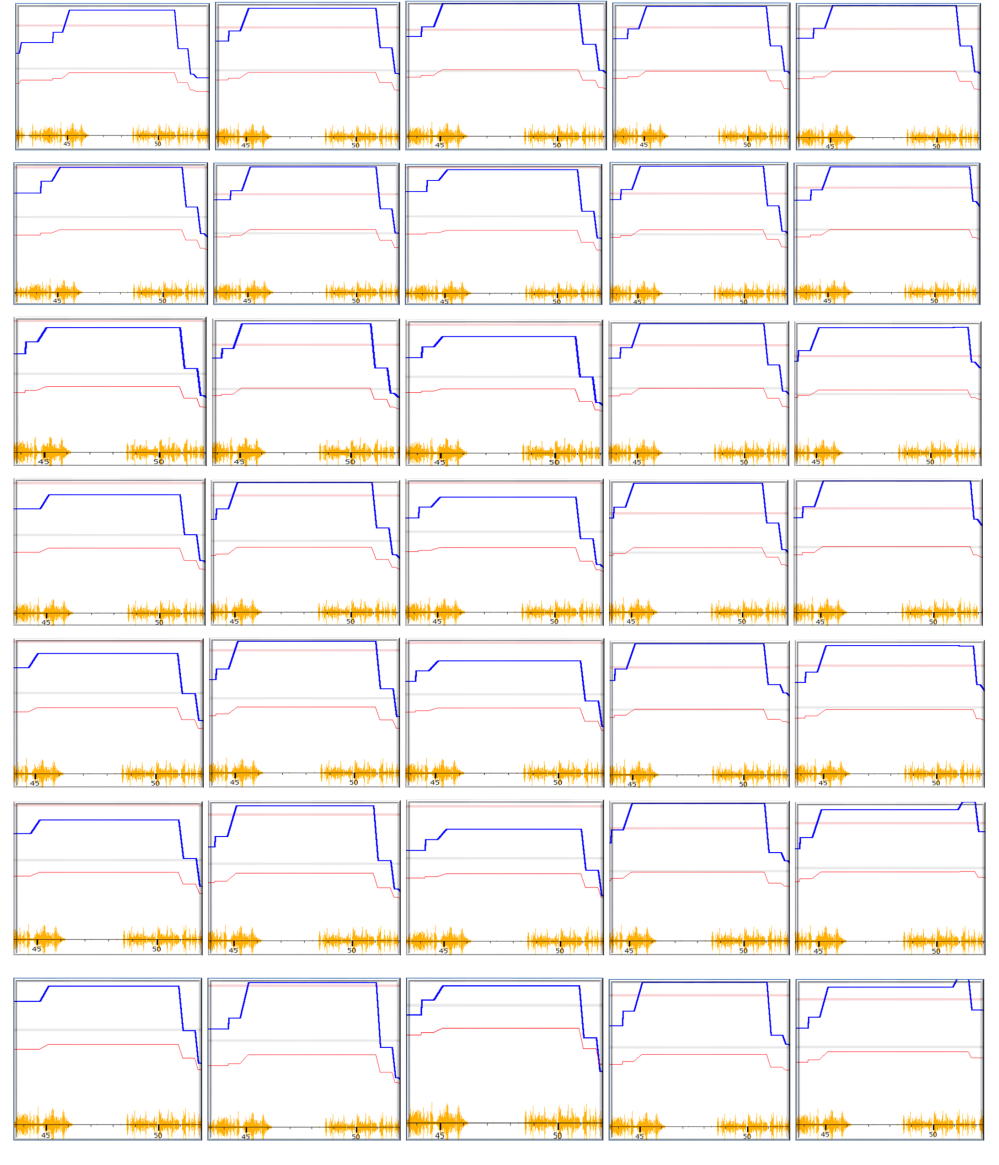
Detection of peak genomic region of QTL of cross sections of femurs in mice using RI strains. The numbers on bottom of each figure indicate the megabases. Pink color lines on top indicate the threshold for significant level. Light grey lines indicate the threshold for suggestive level. From the top to the bottom panel, are the results from reduction of 1, 2, 6, 7, 8, 9, and 10 strains (with actual strain number of 45, 44, 40, 39, 38, 37, and 36 in each test) from a total of 46 strains. These figures show that the peak region of the QTL was mapped to the same location from these tests.

Our result demonstrated that all the tests reproduced the QTL on Chr15 even when 20% of strains were eliminated from the analysis (Figure A in S1 Text). In every analysis, the QTL on Chr 15 reached the detectable level (Fig 2).

The second set of data is from a study for the cerebral cortex volumes bilaterally in 54 BXD RI (GeneNetwork ID 10997) with shrinkage corrected only and data adjusted for shrinkage, age, sex, plane of section [2]. With 2000 permutation tests, LRS criteria for the mapping with the original data of 54 strains at suggestive level = 10.16, significant = 16.02, and highly significant = 18.68. Two QTL loci were detected on Chr6 and Chr11 (Fig 2), with LRS of 14.465 and 14.391, respectively. Similar tests were conducted with reduced number of strains. For both QTLs, the results indicated that all the tests reproduced the QTL even when data of 15% of strains (with data from 45 strains) were eliminated from the analysis (Figure B in S1 Text). In every analysis, the LRS of the QTL on both Chrs were more than the suggestive levels.

In comparison, we tested BMD data from early studies of BMD with less number of strains. The early studies include a study in BMD corrected for whole body weight of both sexes in 2001 (GeneNetwork ID 10443) [3], and bone cross-sectional area of both sexes in 2002 (GeneNetwork ID 10360) [4]. In each of these studies, 20 strains were used for the QTL mapping in the original studies. We sequentially re-produced the QTL maps for each of studies with 2000 permutation tests using GeneNetwork. When 2 strains (10% of the total strains) were taken away from the total RI strains, one of the five replicates was not able to detect the QTL (Figure C in S1 Text). For the data of BMD, a major QTL on Chr X was detected with the data from the original 20 strains but it did not reach the suggestive level in replicate #1. For the data of bone cross-sectional area, two major QTLs on Chr1 and 18 were detected with data from original 20 strains but they did not reach the suggestive level in replicate #4, indicating that the QTL is not reliable or reproducible.

### Determinant of the minimum genomic region of candidate genes of a QTL

Based on the concept of QTL mapping, we made the assumption that, in QTL regions, the most likely determinants of the parameters are those that are the highest. Thus, the genomic parameter at the highest likelihood (the peak region of the QTL) regulates the phenotype. Consequently, the genomic fragment that hosts the QTG is the one that all the sequencing reduction tests map to. We then examined whether the peak genomic regions are located in the same position among the QTL detected by reduced number of strains for the cross section and cerebral cortex volumes.

Fig 2 shows the peak region of QTL for bone cross section tibiae of mice mapped with different numbers of strains, 44, 40, 39, 38, 37, and 36, in comparison to the original 45 strains. Every test of five biological replicates produced the highest LRS region from 45 to 51 megabases (Mb). This region includes the Trps1, which has been identified as the causal gene of QTL for BMD in femurs and tibiae in mice of BXD RI strains [5].

**Fig 3:**
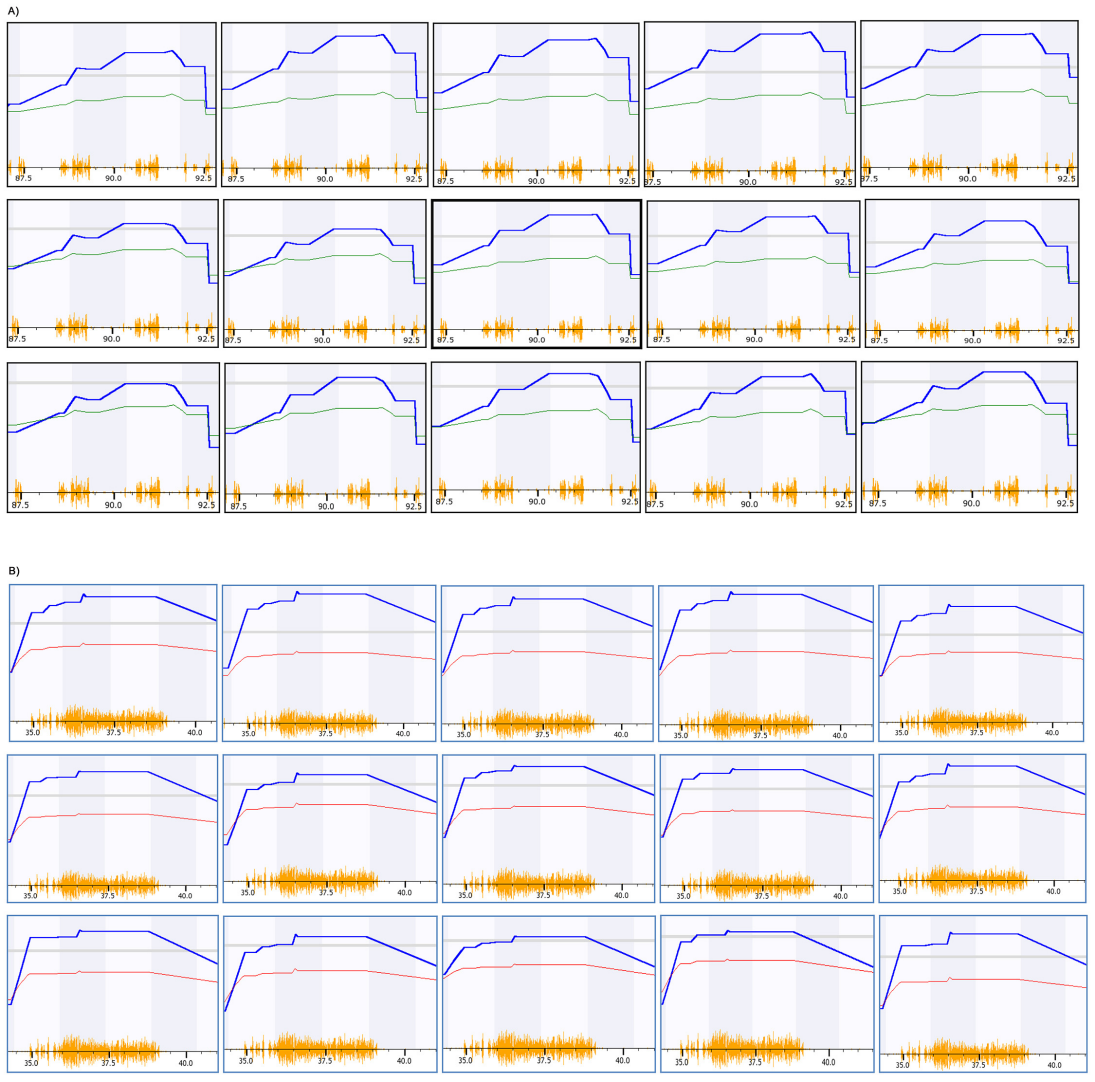
Detection of peak genomic region of QTL of Cerebral cortex volumes in mice using RI strains. The numbers at the bottom of each figure indicate the megabases. Pink color lines on the top indicate the threshold for significant level. Light grey lines indicate the threshold for suggestive level. The upper panel is the peak region for QTL on Chr 6 and the lower panel is for QTL on Chr 11. The three lines of figures from the top to the bottom in each panel are results from elimination of 1, 6 and 9 strains from a total of 54 strains. Therefore, the actual number of mouse strains in these three lines of each of sub-figures (A and B) are 53, 48, and 45, from top to bottom. The peak region of the QTL was mapped to the same location in these tests. A. Test of sample reduction of the QTL on Chr 6. B. Test of sample reduction of the QTL on Chr 11.

Fig 3 shows the peak region of cerebral cortex volumes mapped with a variety of numbers of strains. The initial map with the total of 54 strains identified two QTLs, on Chr 6 and 11. For each QTL, all 15 biological replicates with different number of strains produced the highest LRS on the same genomic region for either QTL on Chr 6 or 11, which was previously reported by Gaglani and colleagues [2]. The QTL on Chr6 was located in a genomic region between 88 and 92.5 Mb and the QTL on Chr11 was between 35 and 40 Mb.

### Additional tests on the ability of determinant of candidate region on 4 additional QTLs

The first set is a study of cocaine response including 54 RI strains (ID 12567). The original mapping with 54 strains detected a QTL on Chr 14. Without initial small number reduction, we directly reduced the strain number into 44, which is more than 15% of strain number reduction. The QTL on Chr 14 is detected from each one of five replicates with data from 44 strains (Figure D-4A in S1 Text). Mapping with reduced number of strains indicated that the candidate gene is located between 92.5 and 100 Mb, which was previously reported by Porcu et al.[6].

The second set of data is from ID 12971 for a study on TNFα cytokine expression level two days after infection with H5N1 influenza A virus [7]. The original analysis includes 46 strains. We reduced the strain number into 39, more than 15% of strain number reduction.

A QTL on Chr 6 was detected with the original 46 strains (Figure D-4B in S1 Text). With the reduced number of strains, the candidate gene is mapped to a region between 7.5 and 15 Mb.

The third set of data is from ID12569, for a study on Deoxycorticosterone (DOC) in cerebral cortex 6 hours after dexamethasone [6]. It was done with 43 RI strains. We reduced the strain number into 36. Two QTL on Chr 4 and 17 were detected with the original 43 strains. With the strain number of 36, both QTL were detected (Figure D-4C in S1 Text). The location of candidate gene on Chr 4 was mapped to between 54 and 63 Mb, and QTL on Chr 17 was mapped to a genomic region between 43.5 and 45 Mb.

The fourth set is ID10866 with 69 strains, a study on the H5N1 influenza A virus survival time. We reduced the strain number into 58, thus, approximately 15% of the reduction of the sample size. The mapping with original 69 strains resulted two QTL on Chr 2 and Chr 17. While the QTL on Chr 17 was detected from every replicate and was mapped to a region between 76 and 79 Mb, QTL on Chr 2 was detected from 4 of 5 replicates detected (Figure D-4D in S1 Text), indicating a less reliability. The genomic region of these two QTLs were reported previously by Boon et al [7].

### Using the same approach, we defined the genomic regions of candidate genes from 16 out of 38 QTL mapped with RI strains

Those QTLs were obtained with relatively large number of RI strains and the candidate genes for many of these QTLs have not been reported. Our data showed that among 38 of traits, 16 of them have reliable reproducibility of the QTL loci in the five biological replicates with reduction of at least 15% of number of strains detected (Table B in S1 Text). It is most likely that the causal candidate genes are within the defined genomic region by these QTL mapping.

**Fig 4:**
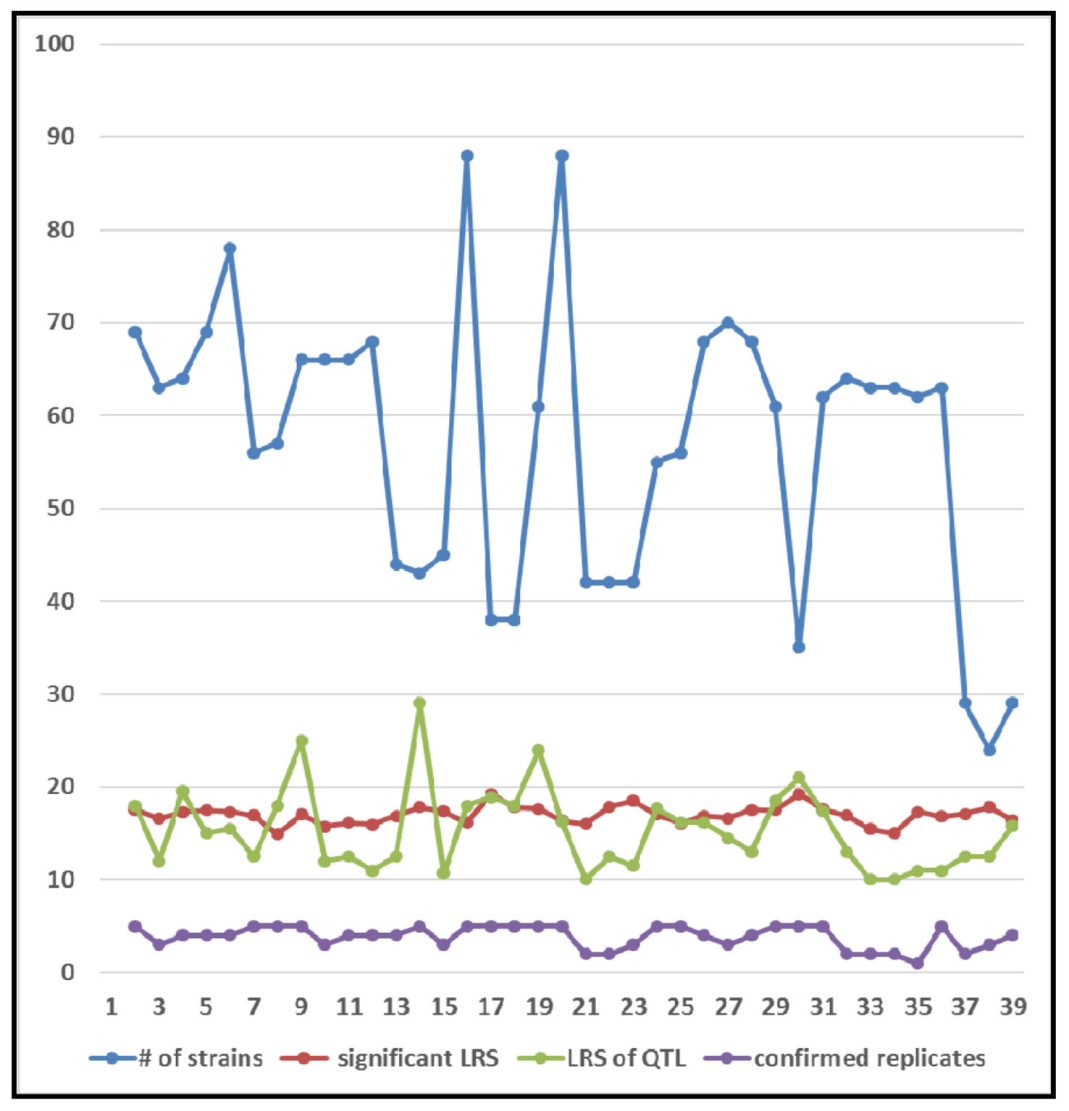
Comparison among number of strains (number of strains), criteria for levels of significant LRS, LRS level of detected QTL, and QTL confirmation in replicates with smaller number of strains.

The analyses also highlight the prerequisite to conduct the test with sequential reduction of strain numbers for these QTL. In general, the higher the LRS or the closer of the QTL LRS to the significant line is, the better the reproducibility of the QTL mapping is (Fig 4). However, there are some exceptions. 1) Some loci with relatively low LRS values have good reproducibility, such as the study of photoreceptor number (ID 11024), cocaine response, locomotion (ID 11472), and ectromelia virus survival (ID 12667). 2) Some loci with high LRS showed poor reproducibility, such as the study of cocaine response (ID 11730), copper level in hippocampus of males (ID 11029) and hippocampus residual weight (ID 13551). Thus, although all the LRS of mappings were generated with 2000 permutation tests, not all the reliability of the mapped QTL were associated with their scores of LRS.

### Confirmation of the strategy in rats

The similar strategy in mouse RI was applied and confirmed for testing of reliability of QTL mapped using rat RI strains. The first set of data is for the phenotype of body weight of mothers (ID 10101). Two major QTLs were detected when the original 26 RI strains were used in the mapping [8]. We reduced the strain number into 24 and 21 for the testing. When the data of 24 strains were used, both QTLs were detected from each one of five replicates. When the data of 21 strains were used, QTL on Chr 2 was detected from each replicate, but the QTL on Chr 16 was detected from three of the five replicates. The genomic region of the candidate genes in the confirmed Chr 2 is the same in the five replicates (Fig 4).

**Fig 5:**
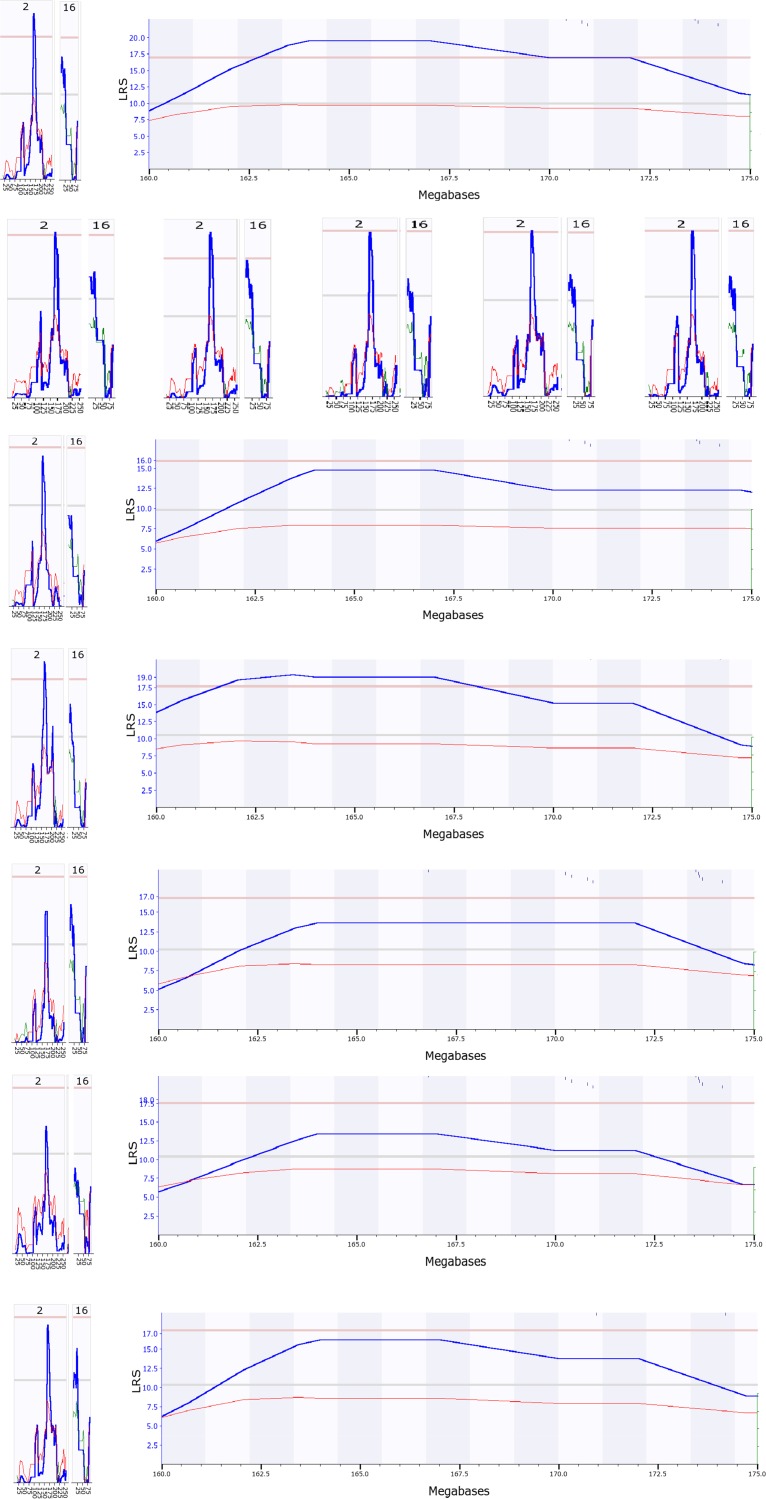
Test of reliability of QTL for the phenotype of body weight of mothers (ID 10101) in rat models by sequential reduction of number of RI strains. The numbers on top of each figure indicate the number of Chrs. The numbers at the bottom of each figure indicate the megabases on the Chrs. Pink color lines indicate the level of significance and grey lines indicate the level of suggestive. The left figure on the top line shows the two major QTLs (Chr 2 and 16) were detected when the original 26 RI strains were used in the mapping. The right figure on the top line shows the genomic region of the peak region of QTL on Chr 2. The five figures on the second line show that both QTLs were detected when the strain number was reduced into 24. The rest figures of five lines (line 3 to 7) show the mapping results of the five replicates when the strain number was reduced into 21. The left figure on each of these five lines show the LRS level of QTL on Chr 2 and 16. QTL on Chr 2 reached the detectable level in each replicate while the QTL on Chr 16 did not reach the detectable level(the grey line) in replicate #1 (line 3) and 4 (line 6). Figures on the right of these five replicates show the peak region of the QTL on Chr 2 was mapped to the same location (between 162.5 Mb and 172.5 Mb).

The second set of data is for the alcohol response of males evaluated by two-bottle choice test (ID10190) [9]. The dataset includes 25 strains. We reduced the strain number into 23 and 21 for the testing. The QTL on Chr 20 was detected from three of five replicates when 23 strains were used for the mapping (Fig 5). When 21 strains were used, the QTL was detected from only two of the five replicates. Thus, the QTL on Chr 20 is not reliable.

### Confirmation of the strategy in plants

**Fig 6:**
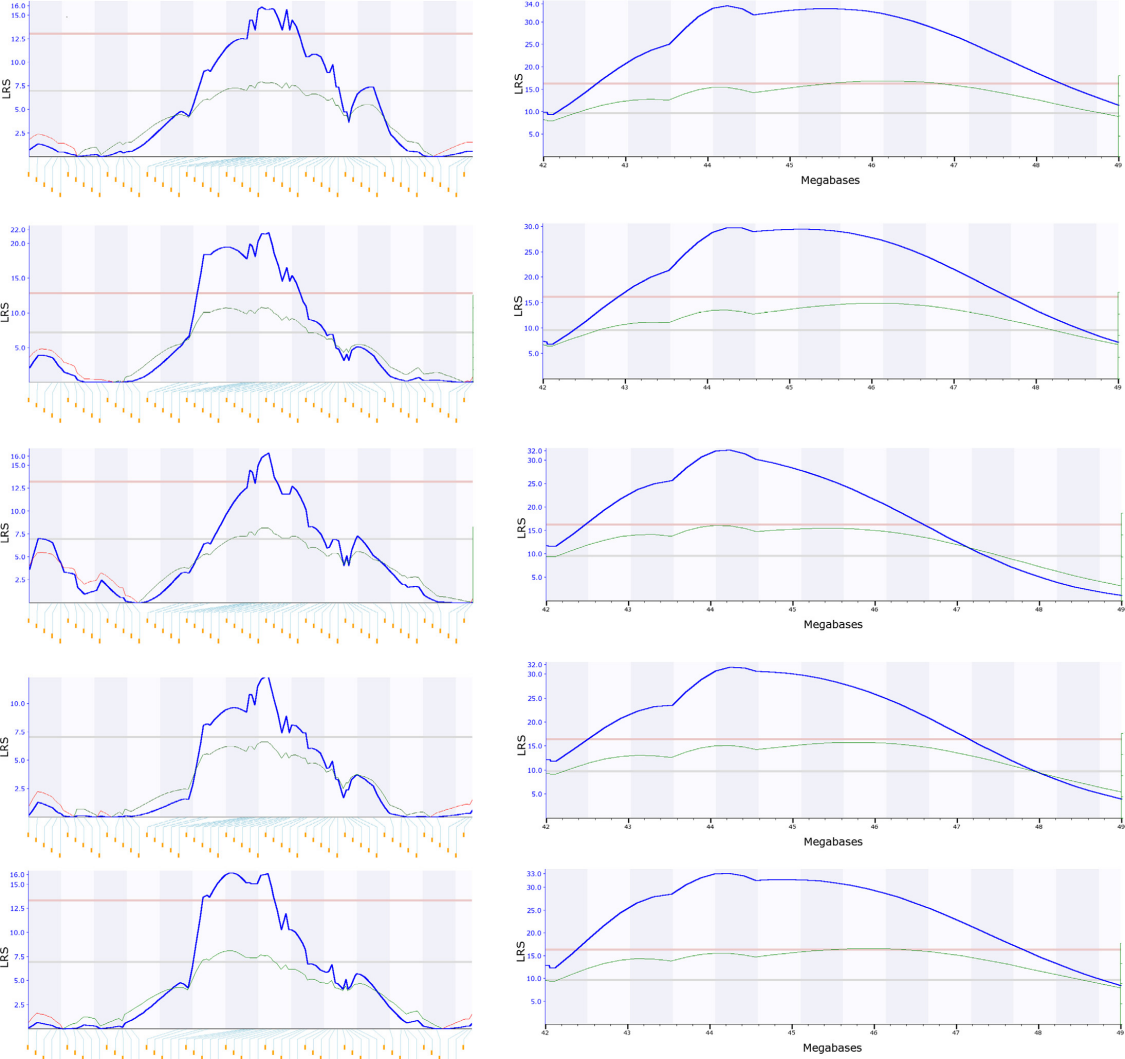
Test of reliability of QTL from two studies of traits of plants by sequential reduction of number of RI strains. The numbers on the left of each figure indicate the LRS. Pink color lines indicate the level of significance and grey lines indicate the level of suggestive. Five figures on the left side are the QTL locations on Chr 6 from a study of Barley phenotype (ID49919). From the top to the bottom are the results from the five replicates, each with the number of strains reduced from original 150 to 125. The yellow lines connected to the blue dots at the bottom of each figure indicate the locations of molecular markers on Chr 6. The figures on the right are the mapping results from a study of soybean phenotype (ID5). The five figures from the top to the bottom are the mapping locations from five replicates, each with the strain number reduced from 143 to 113. The numbers at the bottom of each figure indicate the megabases on the Chrs. Each figure shows the QTL peak region between 44 and 45 Mb on Chr 19.

RI strains have been widely used for the purpose of identification of QTL of economic important traits in plant breeding. We confirmed the strategy for the test of reliability of QTL mapped with plant RI strains. Barley SMP published phenotypes (Database ID 49919) contains 150 RI strains [10]. A QTL was mapped on Chr 6 for the trait of necrotic spotting milky stage. We reduced the number of strains into 125. From each of the five replicates with 125 RI strains, the QTL was mapped to Chr 6 and the peak region is located to the same region (Fig 6).

The data set of soybean (ID 5) node number on main branch has 143 strains. The number of strains was reduced into 113. A highly significant QTL was detected on Chr 19 with the original 143 strains. From each of the five replicates with 113 RI strains, the QTL was mapped to Chr 19 and the peak region is in the same region (Fig 6).

### Applying the sequential reduction of number of strains in F2 mapping

We tested the potential application of the same strategy on QTL mapped with F2 populations. The first set of F2 study for the test (ID 10017) in mice is for Delta Ct numbers of phosphoenopyruvate carboxykinase (NM 011044) measured by quantitative real-time RT-PCR [11–12]. The mapping using the data from the original total of 107 individuals produced two QTLs on Chr 1 and Chr 8. When we conducted the QTL analysis using 96 samples (10% of number reduction of individuals) in each of the 10 replicates, two QTLs were all detected However, when the individuals were reduced to 91 (15% reduction rate), the QTL on Chr 1 did not reach suggestive level in one of the 10 replicates.

The second set of study (ID 10003) in mouse population is the body weight at 8 weeks of age [11]. The data set contains body weight from 100 individuals. Mapping with original data from 100 individuals located two QTLs on Chr 2 and Chr 8. When the number of data was reduced into 85 (15% reduction), two QTL were detected from all the 10 replicates.

We then tested F2 population in rat models. We used the data of gene expression level of eye mRNA (Dataset ID 73, Name: UIOWA Eye mRNA RAE230v2). We first mapped a QTL for regulation of transforming growth factor, beta 1*(Tgfβ1)* expression level using data generated from 120 F2 individuals. Original mapping identified a major QTL on Chr 18. The detection threshold for suggestive and significant LRS levels are 7.25 and 13.39, while the LRS level of QTL on Chr 18 is approximately at 11.5. When data from 18 individuals was taken away, QTL on Chr 18 was detected from each one of 10 replicates. Our second test is on the genetic locus for regulation of procollagen, type III, alpha 1 (Col3a1) expression level. With the 120 samples, the QTL was detected on Chr 13???. The threshold for suggestive and significant LRS are 7.13 and 12.87, while the LRS on Chr 13??? is approximately at 12. When the number of individual data was reduced into 102, the QTL on Chr 16??? was detected from 9 of 10 replicates. These data again indicate that higher LRS in initial mapping does not necessarily mean the QTL is reliable.

Lastly we applied the method to F2 population in the plant. We used a soybean F2 population generated by crossing J12XJ58F2. The population has data from a total of 83 of individuals. We conducted a QTL mapping for the data of yield determination from dataset ID 10010. Original mapping with the data from 83 individuals detected QTL with two locations, one is on Chr 4 and the other is on Chr 23. These two QTLs are detectable from each one of 10 replicates when mapping with data from 73 of individuals, suggesting the strong reliability of these two QTLs.

### Selections of candidate genes from the defined QTL regions

Finally we examined the candidate genes within the genomic regions of three QTLs. We first examined the candidate genes in the QTL region for the bone cross section. From genomic region 45 to 51 megabases (Mb), there are 6 notated genomic elements, four of them are predicted sequences without protein sequences (Figure C in S1 Text). The only two knowns genes are Csmd3 and Trps1. In our previously study, the searching region for the QTL of bone mineral density is from 38 to 52 Mb which contains a total of 73 genetic elements [5].

We next examined the candidate genes in the QTL for cerebral cortex volumes. The QTL on Chr6 was located in a genomic region between 88 and 92.5 Mb. We found 70 candidate genetic elements are within this region. In the previous publication, the location of the QTL was defined as 88 ± 5 Mb which contains 187 genetic elements. Detailed examination narrowed down the candidates into seven (Table D in S1 Text).

We then examined the candidate genes in the genomic region of QTL for body weight of rats on Chr 2. The QTL is defined between 162 and 172.5 Mb which contains 14 genetic elements (Table E in S1 Text). Among these candidates, butyrylcholinesterase (*Bche*) and Sucrase-isomaltase (*Si*) peptidylprolyl isomerase D (*PpiD*) and electron-transferring-flavoprotein dehydrogenase (*Etfd*H) are all well-known for body weight or obesity.

## Discussion

In summary of the procedures above, we propose a procedure to determine the accuracy and precision of a QTL detected as the following:

1. To obtain the location of the peak region of the QTL based on the data of original number of samples.
2. To reduce 10% of the number of samples at least in each one of the five replicates. The eliminated samples in these replicates should be different from each another. Sequential reduction of the number of samples with the order of the replicates is preferred. Data from each one of these replicates should be analyzed for QTL or association program with 2000 permutation tests.
3. If the same QTL to the original number of samples is detected and located on the same location, conduct further tests with five replicates that contain data with reduction of 15% number of samples.
4. If the same QTL to the original number of samples is detected from each one of five replicates with 15% reduction of the samples size, the QTL is considered as reliably. For QTL, it is necessary to remap its specific Chr number and narrow it down to the specific genomic region.

In the case the QTL is not re-detected from replicates with 10% strain number reduction, we consider the original QTL as the fault discovery. In the case the reproducibility is not confirmed at 15% sample reduction, we currently do not have solid evidence to indicate these QTLs are false discovery, however, we believe that other evidence to approve the existence of such a QTL is needed before conducting further studies such as identification of the candidate genes. In the case of large numbers of samples, this may be an indicator for suitability of sub-populations.

While permutation test has been adapted by the QTL analyses and is believed to assure the accuracy of the QTL, it seems it is not capable to detect the influence of many complicated factors such as quality of the data and the strain number limitation of the studies. The strategy of sequential reduction of number of samples provides an experimental tool to test the reliability and the accuracy of the candidate region of the QTL. It takes away the bad curse of the century on the study of complex traits.

The strategy of sequential number reduction of stains paves the way for the utilization of data for study of complex traits using for biomedical agricultural and environmental research. After decades of accumulation, relative larger numbers of RI stain populations in animal models and several major plants have established. The establishment of test of sequential number reduction enables the full exploration of the advantage and potentiality in the usage of samples for the study of complex traits.

We used the five replicates for the sequential reduction of strain number in our study. More replicates can be used according to each individual’s study. However, we believe the number of replicates should not be less than five. With 15% strain number reduction, five replicates eliminate a 75% of the original total number of samples. With 20% of number reduction, every sample was eliminated at least one time. Therefore, we believe that the five replicates is the minimum of number of replicates that are needed for the QTL association confirmation and determination of the candidate region.

Although a small number of strain number usually suggests the less reliability of the QTL, the number limitation in different studies varies. In our study, a large number of samples sometimes detected a non-reliable QTL (Mouse ID12344, 11476, 13587) while a small number of samples sometimes gave a reliable QTL (Mouse ID12667, Rat ID10101). Therefore, test with sequential number reduction of samples is essential for the confirmation of the QTL.

## Conclusion

Based on the data above, we believe that our strategy not only assure the accuracy of QTL location but may also reduce the number of the candidate genes. Therefore, the strategy enhances the ability in identification of causal genes for QTL.

## Supporting Information

S1 Text Detection of QTL for cross section of femurs of mice using RI strains. The numbers on top of each figure indicate the number of chromosome. Pink color lines on top indicate the threshold for significant level. Light grey lines indicate the threshold for suggestive level. Top figures are the mapping results from five replicates of 45 RI strains (with one strain randomly eliminated from the total 46 strains). Middle figures are the mapping results from five replicates of 41 RI strains (with five strain randomly eliminated from the total 46 strains). Bottom figures are the mapping results from five replicates of 36 RI strains (with 10 strain randomly eliminated from the total 46 strains) (Figure A). Detection of QTL for Cerebral cortex volumes of mice using RI strains using sequential reduction of number of strains. The numbers on top of each figure indicate the number of chromosome. Pink color lines on top indicate the threshold for significant level. Light grey lines indicate the threshold for suggestive level. Top figures are the mapping results from five replicates of 53 RI strains (with one strain randomly eliminated from the total 54 strains). Middle figures are the mapping results from five replicates of 48 RI strains (with six strain randomly eliminated from the total 54strains). Bottom figures are the mapping results from five replicates of 35 RI strains (with 9 strain randomly eliminated from the total 54 strains) (Figure B.). Diagnosis of none reproducibility of detection of QTL for bone mineral density and cross sections of mice using small numbers of RI strains using sequential reduction of strain numbers. The numbers on top of each figure indicate the number of chromosome. Pink color lines on top indicate the threshold for significant level. Light grey lines indicate the threshold for suggestive level. Left figures are the QTL detected with the original 20 strains. Right figures are the mapping results from one of five replicates of 18 RI strains (with 2 strain randomly eliminated from the total 20 strains). Upper panel is the QTL detected for the BMD corrected for whole body weight. Lower panel is the QTL detected for the bone cross-sectional area (Figure C). -(S4). Test of reliability of QTL from four additional studies by sequential reduction of number of RI strains. The numbers on top of each figure indicate the number of Chr. The numbers on bottom of each figure indicate the megabases on the Chr. Pink color bars indicate the level of significance and grey bars indicate the level of suggestive. Figure D-4A, 4B, 4C and 4D are for the test for study ID 12657, 12971, 12569 and 10866, respectively. In each test, the far left portion is the QTL detected from the RI strains with the original numbers while the five individual figures on the right are the results of five replicates with reduced number of strains (Figure D). Genomic regions of candidate genes from 38 sets of phenotypic data (Table A). Test of Reliability of SNP detected from GWAS study from 362 samples using strain number reduction (Table B). Candidate genes for size of bone cross section (Table C). Candidate genes for cerebral cortex volumes (Table D). Candidate genes for Rat body weight between 162 and 172.5 MB on chr 2 (Table E).
(DOC)

## Abbreviations

Bche: butyrylcholinesterase
BXD: C57BL/6J (B6) X DBA/2J (D2)
Chr: ChromosomeS
Col3a1: procollagen, type III, alpha 1
Doc: Deoxycorticosterone
EtfdH: electron-transferring-flavoprotein dehydrogenase
LRS: likelihood ratio statistic
Mb: megabases
PpiD: peptidylprolyl isomerase D
QTL: quantitative trait loci
RT-PCR: real time PCR
Si: Sucrase-isomaltase
Tgfβ1: transforming growth factor, beta 1
TNF: tumor necrosis factor
