# Supplementary material for: Self-Confirmation and Ascertainment of the Candidate Genomic Regions of Complex Trait Loci – A None-Experimental Solution
Source: PLoS One. 2016 May 20;11(5):e0153676. doi: 10.1371/journal.pone.0153676 (PMC4874692; doi:10.1371/journal.pone.0153676)
Supplement: S1 Text — The numbers on top of each figure indicate the number of chromosome. Pink color lines on top indicate the threshold for significant level. Light grey lines indicate the threshold for suggestive level. Top figures are the mapping results from five replicates of 45 RI strains (with one strain randomly eliminated from the total 46 strains). Middle figures are the mapping results from five replicates of 41 RI strains (with five strain randomly eliminated from the total 46 strains). Bottom figures are the mapping results from five replicates of 36 RI strains (with 10 strain randomly eliminated from the total 46 strains) (Figure A). Detection of QTL for Cerebral cortex volumes of mice using RI strains using sequential reduction of number of strains. The numbers on top of each figure indicate the number of chromosome. Pink color lines on top indicate the threshold for significant level. Light grey lines indicate the threshold for suggestive level. Top figures are the mapping results from five replicates of 53 RI strains (with one strain randomly eliminated from the total 54 strains). Middle figures are the mapping results from five replicates of 48 RI strains (with six strain randomly eliminated from the total 54strains). Bottom figures are the mapping results from five replicates of 35 RI strains (with 9 strain randomly eliminated from the total 54 strains) (Figure B.). Diagnosis of none reproducibility of detection of QTL for bone mineral density and cross sections of mice using small numbers of RI strains using sequential reduction of strain numbers. The numbers on top of each figure indicate the number of chromosome. Pink color lines on top indicate the threshold for significant level. Light grey lines indicate the threshold for suggestive level. Left figures are the QTL detected with the original 20 strains. Right figures are the mapping results from one of five replicates of 18 RI strains (with 2 strain randomly eliminated from the total 20 strains). Upper panel is t [file pone.0153676.s001.doc]

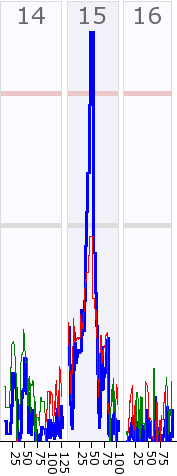

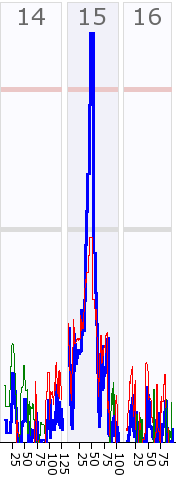

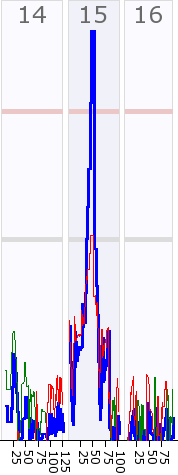

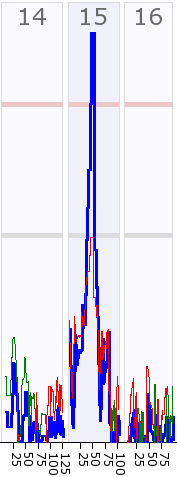

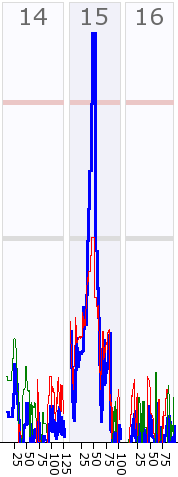


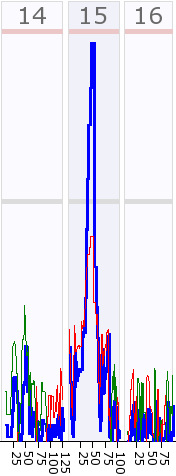

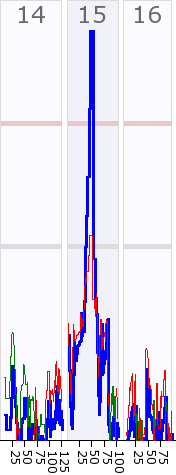

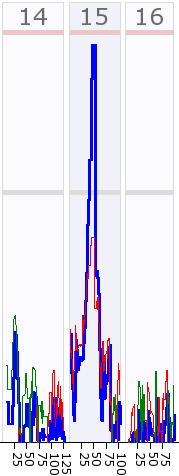

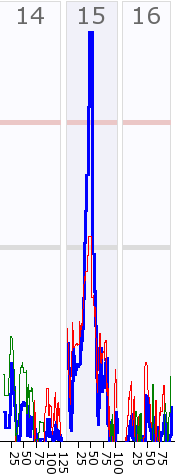

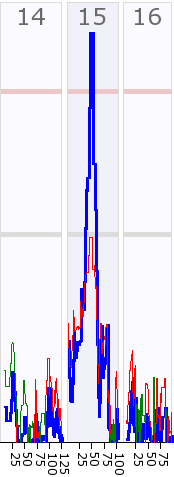


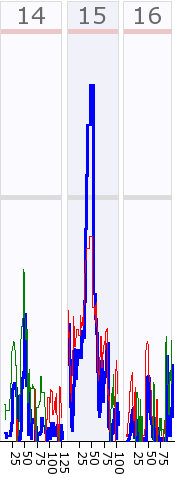

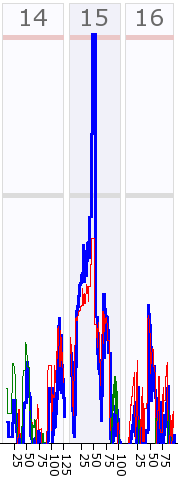

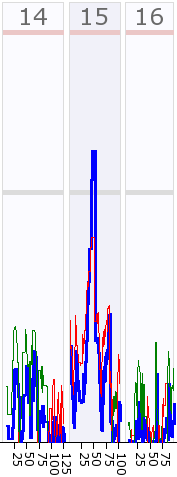

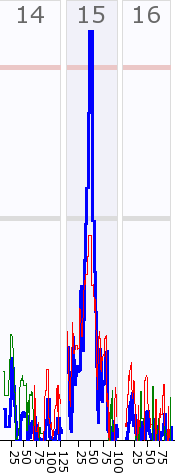

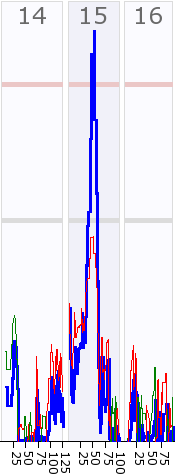


Figure A. Detection of QTL for cross section of femurs of mice using RI strains. The numbers on top of each figure indicate the number of chromosome. Pink color lines on top indicate the threshold for significant level. Light grey lines indicate the threshold for suggestive level. Top figures are the mapping results from five replicates of 45 RI strains (with one strain randomly eliminated from the total 46 strains). Middle figures are the mapping results from five replicates of 41 RI strains (with five strain randomly eliminated from the total 46 strains). Bottom figures are the mapping results from five replicates of 36 RI strains (with 10 strain randomly eliminated from the total 46 strains).


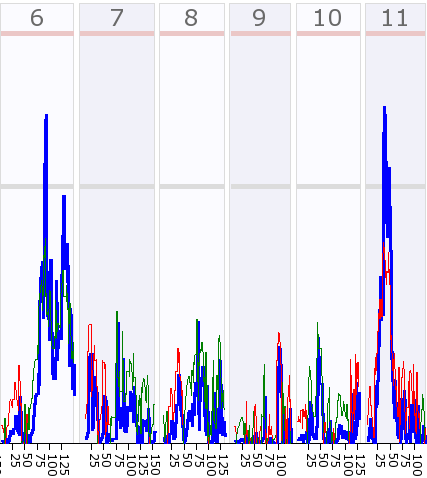

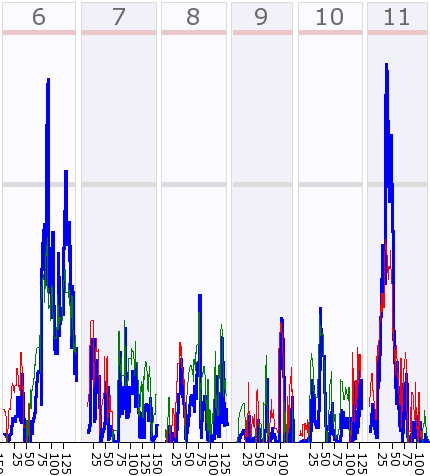

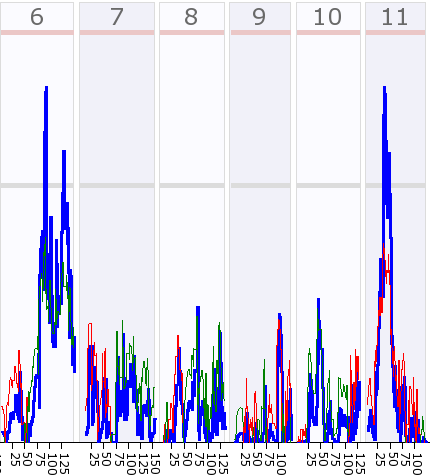

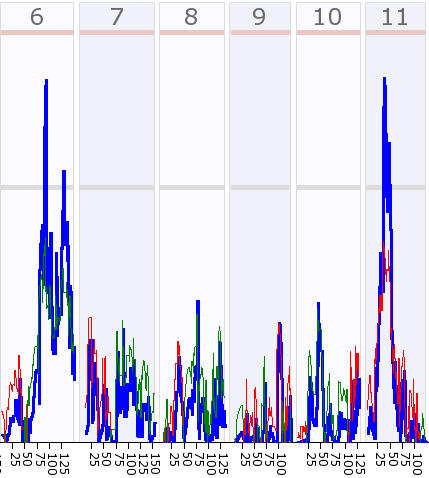

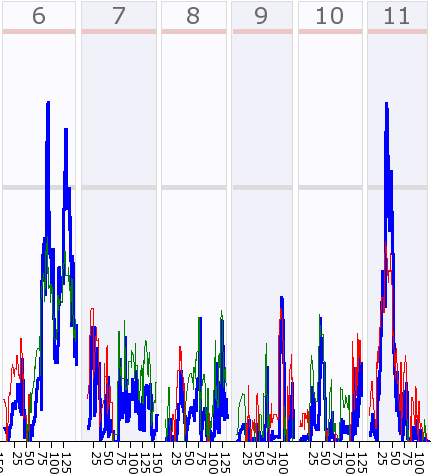


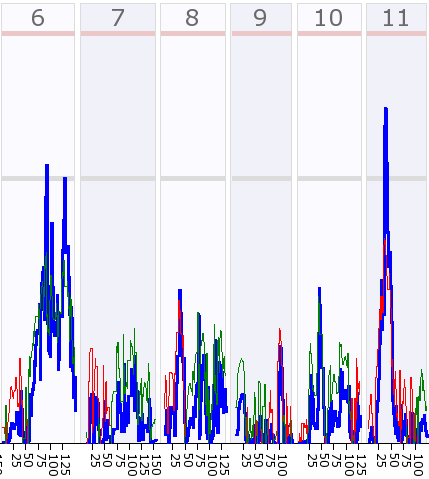

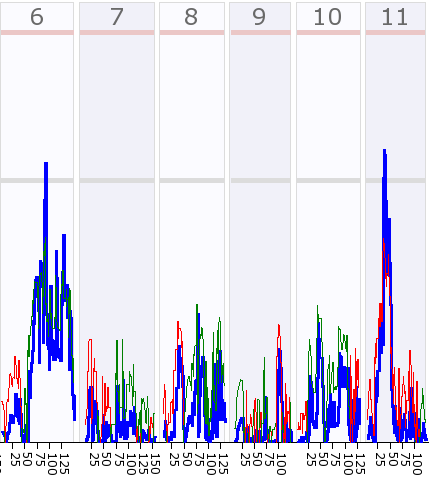

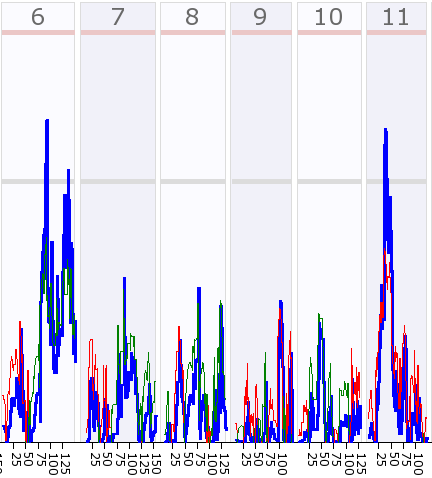

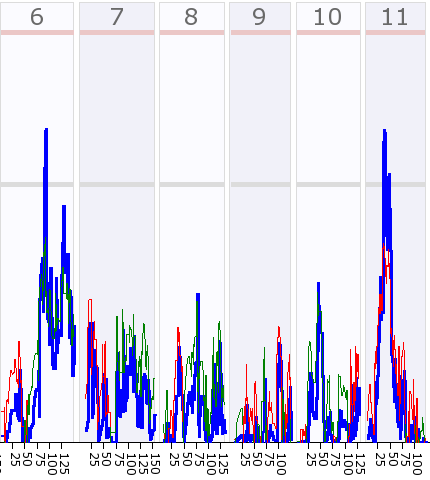

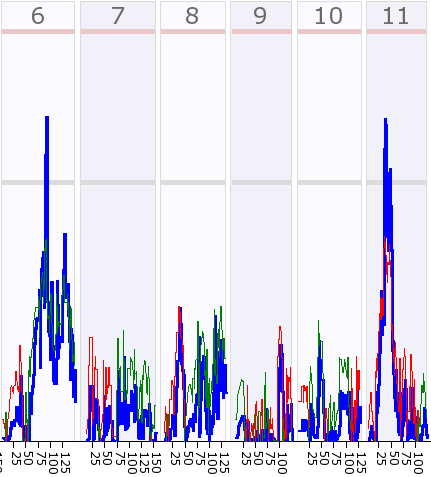


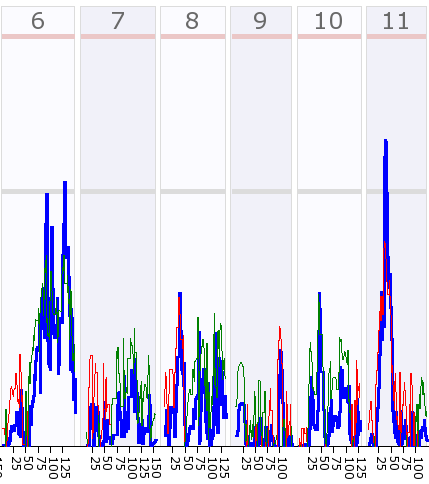

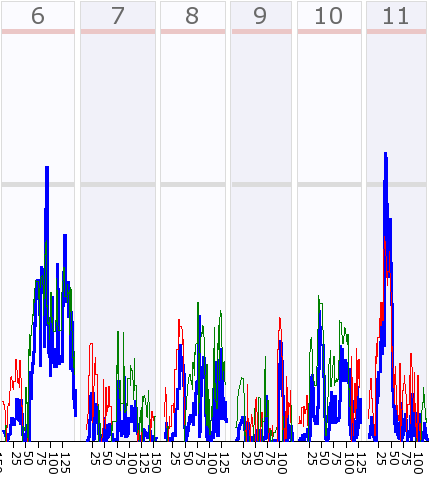

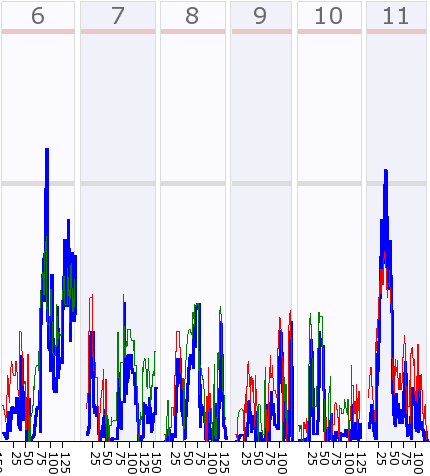

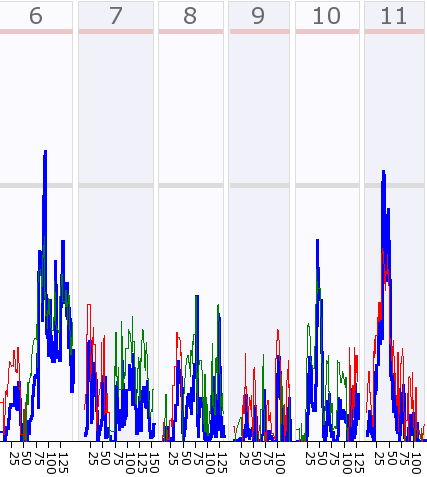

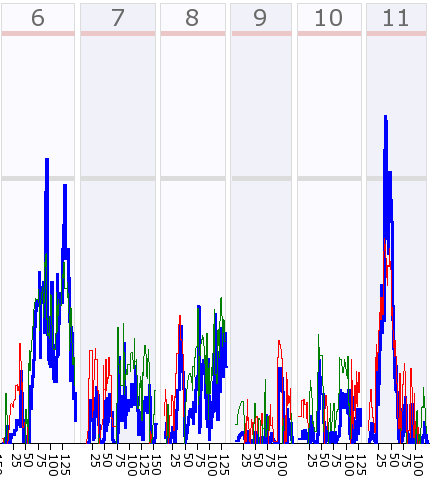


Figure B. Detection of QTL for Cerebral cortex volumes of mice using RI strains using sequential reduction of number of strains. The numbers on top of each figure indicate the number of chromosome. Pink color lines on top indicate the threshold for significant level. Light grey lines indicate the threshold for suggestive level. Top figures are the mapping results from five replicates of 53 RI strains (with one strain randomly eliminated from the total 54 strains). Middle figures are the mapping results from five replicates of 48 RI strains (with six strain randomly eliminated from the total 54strains). Bottom figures are the mapping results from five replicates of 35 RI strains (with 9 strain randomly eliminated from the total 54 strains).


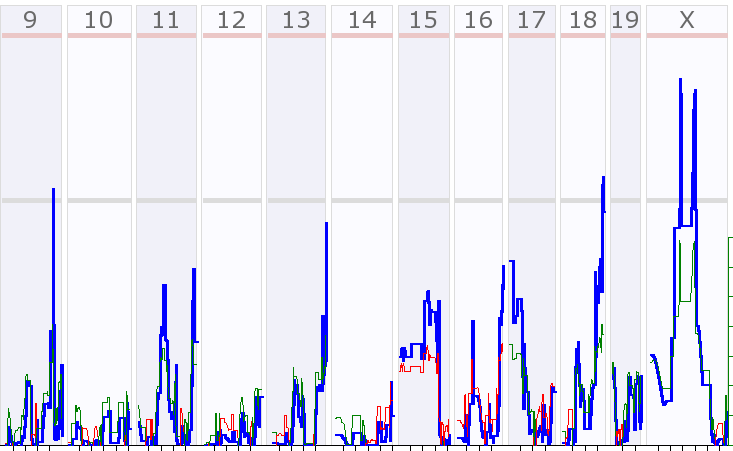

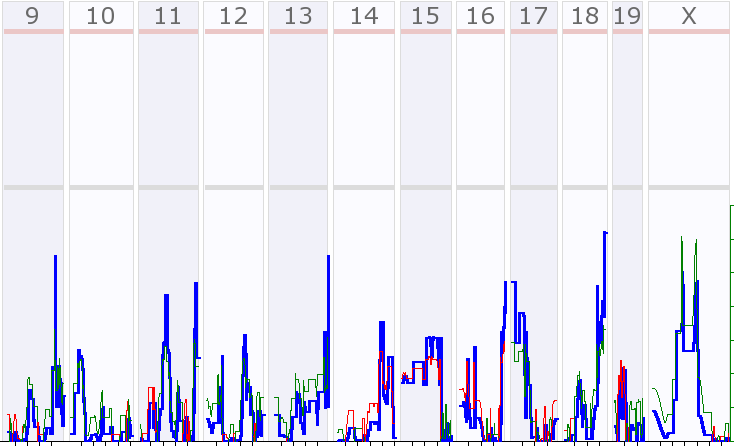


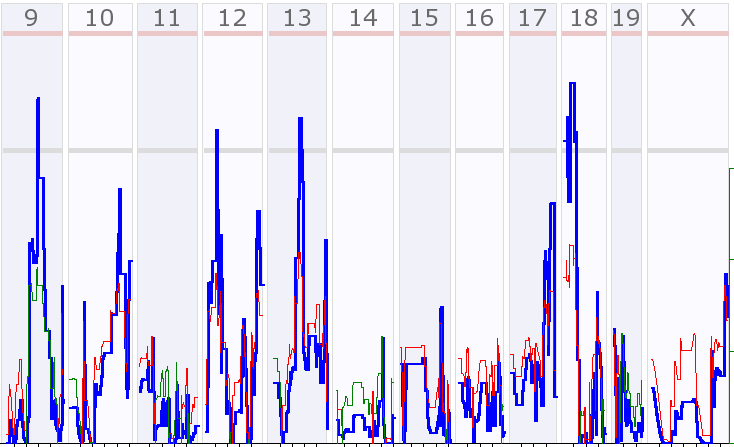

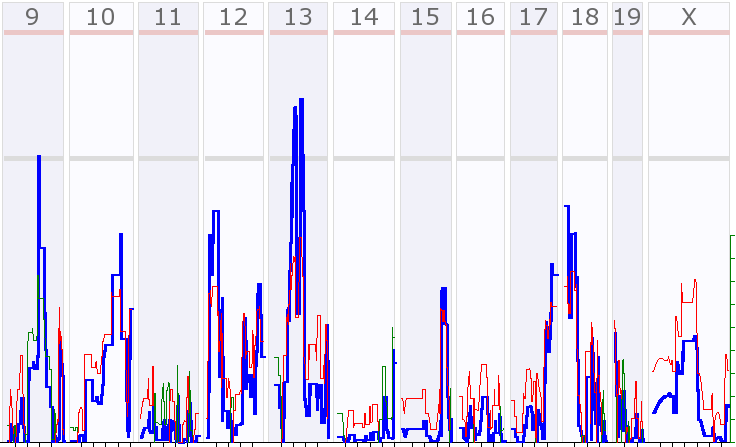


Figure C. Diagnosis of none reproducibility of detection of QTL for bone mineral density and cross sections of mice using small numbers of RI strains using sequential reduction of strain numbers . The numbers on top of each figure indicate the number of chromosome. Pink color lines on top indicate the threshold for significant level. Light grey lines indicate the threshold for suggestive level. Left figures are the QTL detected with the original 20 strains. Right figures are the mapping results from one of five replicates of 18 RI strains (with 2 strain randomly eliminated from the total 20 strains). Upper panel is the QTL detected for the BMD corrected for whole body weight. Lower panel is the QTL detected for the bone cross-sectional area.


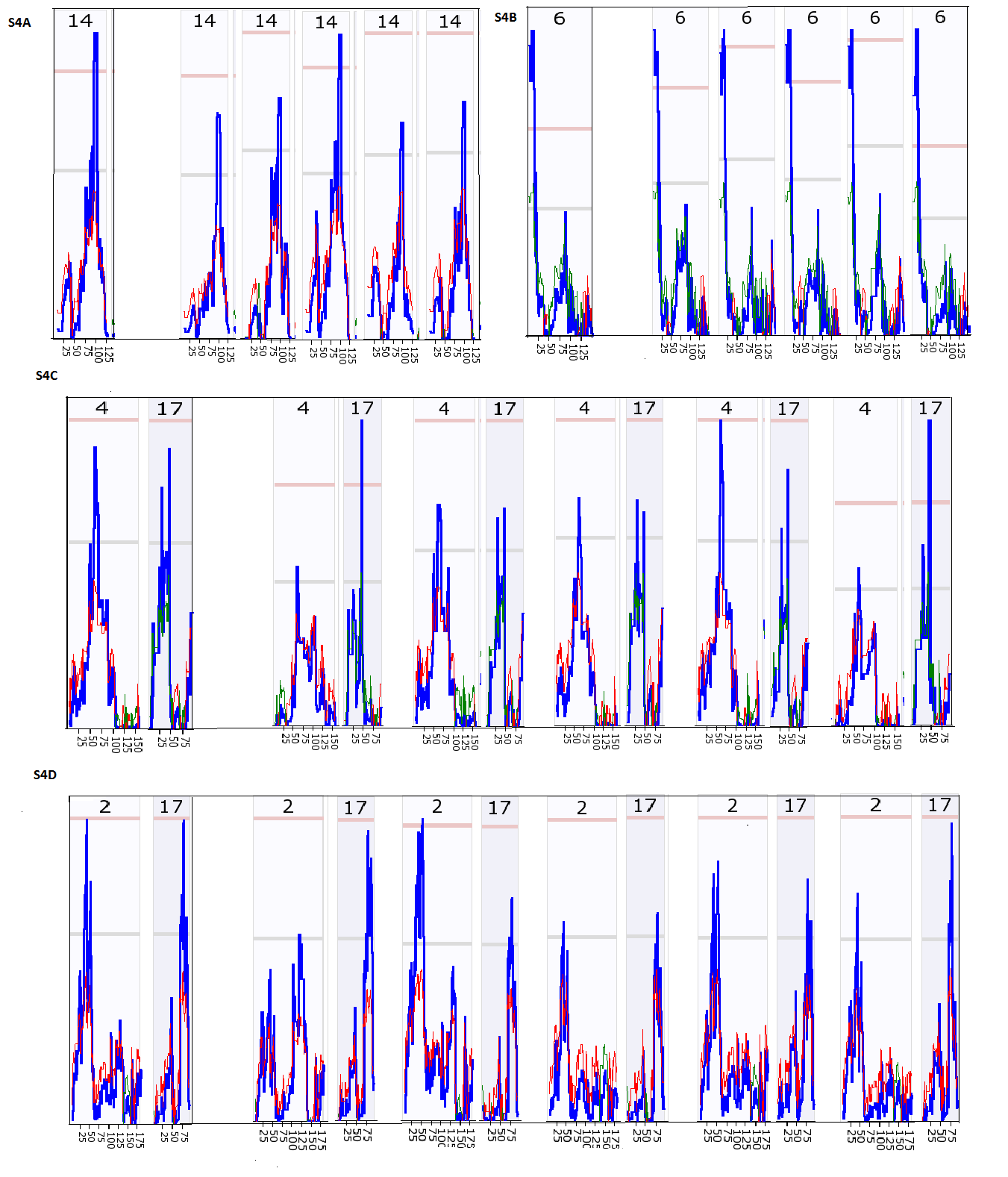


Figure D. -(S4). Test of reliability of QTL from four additional studies by sequential reduction of number of RI strains. The numbers on top of each figure indicate the number of Chr. The numbers on bottom of each figure indicate the megabases on the Chr. Pink color bars indicate the level of significance and grey bars indicate the level of suggestive. Figure S4A, B, C and D are for the test for study ID 12657, 12971, 12569 and 10866, respectively. In each test, the far left portion is the QTL detected from the RI strains with the original numbers while the five individual figures on the right are the results of five replicates with reduced number of strains.

Table A. Genomic regions of candidate genes from 38 sets of phenotypic data

| Trait/ID | # of original/ reduced strains | Original significant/suggestive LRS | Detected Chromosome /LRS with original number of strains | confirmed/none confirmed replicates | Number of genes and potential candidates |
| --- | --- | --- | --- | --- | --- |
| H5N1 influenza A virus mortality rate 30 days after infection/10865 | 69/58 | 17.52/10.85 | 2/18.0 | 5/0 | Lrp1b |
| Cocaine response, locomotion from 45-60 min after first injection in an activity chamber for males/11476 | 63/53 | 16.6/10.43 | 1/12 | 3/2 | 32 (56-59 mb) |
| Cocaine response, locomotion from 0-15 min after first injection in an activity chamber for females/11730 | 64/53 | 17.29/10.63 | 15/19.5 | *4/1* | 55 (35-43 mb) |
| Photoreceptor density (rods and cones) in young adults/10891 | 69/58 | 17.46/10.71 | 4/15 | 4/1 | 34 (57.2-60.2 mb) |
| Central nervous system, development, morphology/11023 | 78/66 | 17.31/10.63 | 5/15.5 | 4/1 | 37 (104.1-106.8 mb) |
| Photoreceptor number/11024 | 56/48 | 16.92/10.70 | 6/12.5 | **5/0** | 12 (15-18 mb) |
| Soleus-femoral muscle attachment anomaly [frequency]/11033 | 57/48 | 14.86/9.64 | 14/18 | 5/0 | 29 (59-62mb) |
| Mean primary interlick interval for water over 20 min/12297 | 66/58 | 17.02/10/51 | 1/25 | 5/0 | 130 (172-178 mb) |
| Anxiety assay, baseline untreated control, activity in closed quadrants/12344 | 66/57 | 15.73/10.07 | 19/12 | 3/2 | 17 (14.8-18.4 mb) |
| Anxiety assay, restraint and ethanol (RSE group), time in open quadrants/12436 | 66/57 | 16.11/10.35 | 8/12.5 | 4/1 | 163 (70.0-76.0 mb) |
| Anxiety assay, restraint stress + saline treated (RSS group), activity in closed quadrants/12463 | 68/58 | 15.94/10.02 | 5/11 | 4/1 | 155 (120-128 mb) |
| Cerebellar Purkinje cell number, with Abercrombie correction/12485 | 44/37 | 16.89/10.66 | 14/12.5 | 4/1 | 104 (55-58 mb) |
| Deoxycorticosterone (DOC) in cerebral cortex/12568 | 43/36 | 17.84/10.57 | 4/29 | 5/0 | 143 (46-63 mb) |
| consumption of 15% ethanol using two-bottle choice system/12577 | 45/37 | 17.37/10.68 | 9/10.70 | 3/2 | 56 (25-35 mb) |
| Brain weight, corrected only for dissection error/12659 | 88/75 | 16.14/10.12 | 19/18 | 5/0 | 229 (9-15 mb) |
| Ectromelia virus survival/12667 | 38/32 | 19.24/11.27 | 3/18.9 | **5/0** | 11(15.8-18.8) |
| Ectromelia virus formation of secondary dermal lesions/12668 | 38/32 | 17.81/10.70 | 12/18 | 5/0 | 79 (15-30 mb) |
| Ectromelia virus mortality score/12683 | 61/52 | 17.64/10.78 | 6/24 | 5/0 | 166 (128-138 mb) |
| Body weight/12685 | 88/75 | 16.36/10.24 | 11/16.3 | 5/0 | 216 (73-83 mb) |
| Body weight at bone densitometry assay/12911 | 42/35 | 15.99/9.86 | 9/10.01 | 2/3 | 79 (45-51mb) |
| blood alcohol concentration (BAC) 1-3 hr after gavage of young adult females/12981 | 42/35 | 17.86/10.88 | 17/12.5 | 2/3 | 62(40-46 mb) |
| blood alcohol concentration (BAC) 24 hr after gavage of young adult females/12983 | 42/35 | 18.53/11.04 | 2/11.50 | 3/2 | 133 (67-80mb) |
| Dorsal striatum (caudate putamen) residual volume/13548 | 55/46 | 17.03/10.55 | 6/17.7 | 5/0 | 212 (77-93 mb) |
| Cerebral cortex (neocortex) residual volume/13550 | 56/46 | 16.09/10.07 | 11/16.20 | 5/0 | 16 (35-42 mb) |
| Hippocampus residual weight/13551 | 68/57 | 16.84/10.33 | 1/16.2 | 4/1 | 82 (141-157 mb) |
| Cerebellum residual weight, repeat measurement/13587 | 70/58 | 16.61/10.33 | 1/14.5 | 3/2 | 153 (174-185 mb) |
| Reproductive system/14781 | 68/57 | 17.52/10.84 | 10/13 | 4/1 | 72 (20-28mb) |
| Trait_1_GENEX_CB_BXD/16180 | 61/52 | 17.51/10.60 | 18/18.5 | 5/0 | 52 (47-57 mb) |
| Herpes simplex virus type 1, virulence following corneal infection/16185 | 35/30 | 19.23/11.28 | 16/21 | 5/0 | 79 (83-90 mb) |
| Ethanol response, locomotor activity after injection/11960 | 62/52 | 17.58/10.81 | 9/17.4 | 5/0 | 68 (73-82 mb) |
| Cocaine response, locomotion/11983 | 64/53 | 16.93/10.56 | 15/13 | 2/3 | 55 (35-43 mb) |
| Cocaine response, conditioned place preference/12009 | 63/52 | 15.44/9.98 | 6/10 | 2/3 | 127 (127-134 mb) |
| Cocaine response, conditioned place preference/12012 | 63/52 | 15.00/9.74 | 5/10 | 2/3 | 88 (106-111mb) |
| Ethanol response , locomotor activity/11963 | 62/52 | 17.29/10.65 | 14/11 | 1/4 | 45 (47-50 mb) |
| Cocaine response, locomotion /11472 | 63/53 | 16.78/10.42 | 1/11 | **5/0** | 73 (54-60 mb) |
| Zinc level in hippocampus/11031 | 29/24 | 17.09/10.33 | 9/12.5 | 2/3 | - |
| Transferrin saturation/12546 | 24/21 | 17.80/10.51 | 1/12.5 | 3/2 | 42 (110-145 mb) |
| Copper level in hippocampus of males/11029 | 29/24 | 16.42/9.91 | 9/15.8 | 4/1 | 38 (39.5-41.5 |

Table B. Test of Reliability of SNP detected from GWAS study from 362 samples using sample size reduction

| SNP | rs10928663 | rs807774 | rs807780 | rs2081967 | rs10515720 | rs1934317 |
| --- | --- | --- | --- | --- | --- | --- |
| Chromosome | 2 | 4 | 4 | 5 | 5 | 6 |
| **Original –LOG value** | **6.49621** | **6.33555** | **6.62069** | **6.20433** | **6.20544** | **6.35174** |
| –LOG value in test #1 | 6.78701 | 5.39416 | 5.66817 | 6.37100 | 6.41229 | 6.15752 |
| –LOG value in test #2 | 6.37407 | 5.73119 | 5.87387 | 5.59024 | 5.71287 | 5.71019 |
| –LOG value in test #3 | - | - | - | - | - | - |
| –LOG value in test #4 | 6.16279 | 6.18535 | 6.31722 | 6.72262 | 6.99525 | 5.90101 |
| –LOG value in test #5 | 5.99957 | 6.87354 | 7.18789 | 6.13888 | 6.07572 | 7.28567 |
| –LOG value in test #6 | 6.95821 | 6.26664 | 6.63941 | 7.06013 | 6.94615 | 6.81135 |
| –LOG value in test #7 | 6.52651 | 8.17160 | 8.60223 | 6.09572 | 5.44249 | 6.79210 |
| –LOG value in test #8 | 6.88739 | 5.50710 | 5.77109 | 5.84680 | 6.31867 | 5.97143 |
| –LOG value in test #9 | 3.03391 | 3.66294 | 3.75007 | 3.87550 | 3.98172 | 5.45792 |
| –LOG value in test #10 | 3.79344 | - | - | - | - | - |

Table C. Candidate genes for size of bone cross section

| *Index* | *Symbol* | *Mb Start (mm9)* | *Length (Kb)* | *SNP Count* | *SNP Density* | *Avg Expr* | *Human Chr* | *Mb Start (hg19)* | *Gene Description*  [***\***](javascript:xmlhttpPost('/webqtl/main.py?FormID=AJAX_table', 'sortable', 'sort=description&order=up&file=Mapping_ayaoyYvH&tableID=sortable&addIndex=0&hiddenColumns=')) | *PolymiRTS Database* [*>>*](http://compbio.uthsc.edu/miRSNP/) | *Gene Weaver Info Content* [*>>*](http://geneweaver.org/) |
| --- | --- | --- | --- | --- | --- | --- | --- | --- | --- | --- | --- |
| 1 | [1700022A22Rik](http://www.ncbi.nlm.nih.gov/entrez/query.fcgi?db=gene&cmd=Retrieve&dopt=Graphics&list_uids=71120) **[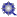](http://www.genenetwork.org/webqtl/main.py?cmd=sch&gene=1700022A22Rik&alias=1)** | [46.156130](http://genome.ucsc.edu/cgi-bin/hgTracks?clade=vertebrate&org=Mouse&db=mm9&position=chr15%3A46156130-46205445&pix=620&Submit=submit) | [49.315](javascript:centerIntervalMapOnRange2('15', 46.1511985, 46.2103765, document.changeViewForm)) | [17](http://www.genenetwork.org/webqtl/main.py?FormID=snpBrowser&chr=15&start=46.15613&end=46.205445&geneName=1700022A22Rik&s1=2&s2=3) | 0.344723 | -- | -- | [--](http://www.genenetwork.org/webqtl/--) | RIKEN cDNA 1700022A22 gene |  |  |
| 2 | [4930523O13Rik](http://www.ncbi.nlm.nih.gov/entrez/query.fcgi?db=gene&cmd=Retrieve&dopt=Graphics&list_uids=74726) **[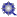](http://www.genenetwork.org/webqtl/main.py?cmd=sch&gene=4930523O13Rik&alias=1)** | [46.267774](http://genome.ucsc.edu/cgi-bin/hgTracks?clade=vertebrate&org=Mouse&db=mm9&position=chr15%3A46267774-46327695&pix=620&Submit=submit) | [59.921](javascript:centerIntervalMapOnRange2('15', 46.2617819, 46.3336871, document.changeViewForm)) | [2](http://www.genenetwork.org/webqtl/main.py?FormID=snpBrowser&chr=15&start=46.267774&end=46.327695&geneName=4930523O13Rik&s1=2&s2=3) | 0.033377 | -- | -- | [--](http://www.genenetwork.org/webqtl/--) | RIKEN cDNA 4930523O13 gene |  |  |
| 3 | [4930548G14Rik](http://www.ncbi.nlm.nih.gov/entrez/query.fcgi?db=gene&cmd=Retrieve&dopt=Graphics&list_uids=75281) **[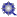](http://www.genenetwork.org/webqtl/main.py?cmd=sch&gene=4930548G14Rik&alias=1)** | [46.454850](http://genome.ucsc.edu/cgi-bin/hgTracks?clade=vertebrate&org=Mouse&db=mm9&position=chr15%3A46454850-46471404&pix=620&Submit=submit) | [16.554](javascript:centerIntervalMapOnRange2('15', 46.4531946, 46.4730594, document.changeViewForm)) | [1](http://www.genenetwork.org/webqtl/main.py?FormID=snpBrowser&chr=15&start=46.45485&end=46.471404&geneName=4930548G14Rik&s1=2&s2=3) | 0.060408 | -- | -- | [--](http://www.genenetwork.org/webqtl/--) | RIKEN cDNA 4930548G14 gene |  |  |
| 4 | [Csmd3](http://www.ncbi.nlm.nih.gov/entrez/query.fcgi?db=gene&cmd=Retrieve&dopt=Graphics&list_uids=239420) **[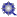](http://www.genenetwork.org/webqtl/main.py?cmd=sch&gene=Csmd3&alias=1)** | [47.412183](http://genome.ucsc.edu/cgi-bin/hgTracks?clade=vertebrate&org=Mouse&db=mm9&position=chr15%3A47412183-48623535&pix=620&Submit=submit) | [1211.352](javascript:centerIntervalMapOnRange2('15', 47.2910478, 48.7446702, document.changeViewForm)) | [66](http://www.genenetwork.org/webqtl/main.py?FormID=snpBrowser&chr=15&start=47.412183&end=48.623535&geneName=Csmd3&s1=2&s2=3) | 0.054485 | -- | 8 | [113.304336](http://genome.ucsc.edu/cgi-bin/hgTracks?clade=vertebrate&org=Human&db=hg17&position=chr8:113304336-114518418) | CUB and Sushi multiple dom... |  |  |
| 5 | [Trps1](http://www.ncbi.nlm.nih.gov/entrez/query.fcgi?db=gene&cmd=Retrieve&dopt=Graphics&list_uids=83925) **[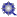](http://www.genenetwork.org/webqtl/main.py?cmd=sch&gene=Trps1&alias=1)** | [50.486304](http://genome.ucsc.edu/cgi-bin/hgTracks?clade=vertebrate&org=Mouse&db=mm9&position=chr15%3A50486304-50721587&pix=620&Submit=submit) | [235.283](javascript:centerIntervalMapOnRange2('15', 50.4627757, 50.7451153, document.changeViewForm)) | [492](http://www.genenetwork.org/webqtl/main.py?FormID=snpBrowser&chr=15&start=50.486304&end=50.721587&geneName=Trps1&s1=2&s2=3) | 2.091099 | -- | 8 | [116.489899](http://genome.ucsc.edu/cgi-bin/hgTracks?clade=vertebrate&org=Human&db=hg17&position=chr8:116489899-116750429) | trichorhinophalangeal synd... |  |  |
| 6 | [5330433J24Rik](http://www.ncbi.nlm.nih.gov/entrez/query.fcgi?db=gene&cmd=Retrieve&dopt=Graphics&list_uids=78276) **[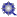](http://www.genenetwork.org/webqtl/main.py?cmd=sch&gene=5330433J24Rik&alias=1)** | [50.572009](http://genome.ucsc.edu/cgi-bin/hgTracks?clade=vertebrate&org=Mouse&db=mm9&position=chr15%3A50572009-50573919&pix=620&Submit=submit) | [1.910](javascript:centerIntervalMapOnRange2('15', 50.571818, 50.57411, document.changeViewForm)) | [4](http://www.genenetwork.org/webqtl/main.py?FormID=snpBrowser&chr=15&start=50.572009&end=50.573919&geneName=5330433J24Rik&s1=2&s2=3) | 2.094241 | -- | -- | [--](http://www.genenetwork.org/webqtl/--) | RIKEN cDNA 5330433J24 gene |  |  |

Table D. Candidate genes for cerebral cortex volumes

| *Index*  ***[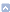](javascript:xmlhttpPost('/webqtl/main.py?FormID=AJAX_table',%20'sortable',%20'sort=index&order=up&file=Mapping_CxSAkzH6&tableID=sortable&addIndex=0&hiddenColumns='))[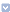](javascript:xmlhttpPost('/webqtl/main.py?FormID=AJAX_table',%20'sortable',%20'sort=index&order=down&file=Mapping_CxSAkzH6&tableID=sortable&addIndex=0&hiddenColumns='))*** | *Symbol*  ***[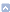](javascript:xmlhttpPost('/webqtl/main.py?FormID=AJAX_table',%20'sortable',%20'sort=symbol&order=up&file=Mapping_CxSAkzH6&tableID=sortable&addIndex=0&hiddenColumns='))[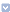](javascript:xmlhttpPost('/webqtl/main.py?FormID=AJAX_table',%20'sortable',%20'sort=symbol&order=down&file=Mapping_CxSAkzH6&tableID=sortable&addIndex=0&hiddenColumns='))*** | *Mb Start (mm9)*  ***[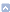](javascript:xmlhttpPost('/webqtl/main.py?FormID=AJAX_table',%20'sortable',%20'sort=mb_start_mm9&order=up&file=Mapping_CxSAkzH6&tableID=sortable&addIndex=0&hiddenColumns='))[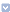](javascript:xmlhttpPost('/webqtl/main.py?FormID=AJAX_table',%20'sortable',%20'sort=mb_start_mm9&order=down&file=Mapping_CxSAkzH6&tableID=sortable&addIndex=0&hiddenColumns='))*** | *Length (Kb)*  ***[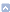](javascript:xmlhttpPost('/webqtl/main.py?FormID=AJAX_table',%20'sortable',%20'sort=length&order=up&file=Mapping_CxSAkzH6&tableID=sortable&addIndex=0&hiddenColumns='))[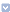](javascript:xmlhttpPost('/webqtl/main.py?FormID=AJAX_table',%20'sortable',%20'sort=length&order=down&file=Mapping_CxSAkzH6&tableID=sortable&addIndex=0&hiddenColumns='))*** | *SNP Count*  ***[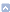](javascript:xmlhttpPost('/webqtl/main.py?FormID=AJAX_table',%20'sortable',%20'sort=snp_count&order=up&file=Mapping_CxSAkzH6&tableID=sortable&addIndex=0&hiddenColumns='))[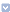](javascript:xmlhttpPost('/webqtl/main.py?FormID=AJAX_table',%20'sortable',%20'sort=snp_count&order=down&file=Mapping_CxSAkzH6&tableID=sortable&addIndex=0&hiddenColumns='))*** | *SNP Density*  ***[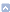](javascript:xmlhttpPost('/webqtl/main.py?FormID=AJAX_table',%20'sortable',%20'sort=snp_density&order=up&file=Mapping_CxSAkzH6&tableID=sortable&addIndex=0&hiddenColumns='))[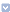](javascript:xmlhttpPost('/webqtl/main.py?FormID=AJAX_table',%20'sortable',%20'sort=snp_density&order=down&file=Mapping_CxSAkzH6&tableID=sortable&addIndex=0&hiddenColumns='))*** | *Avg Expr* | *Human Chr*  ***[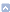](javascript:xmlhttpPost('/webqtl/main.py?FormID=AJAX_table',%20'sortable',%20'sort=human_chr&order=up&file=Mapping_CxSAkzH6&tableID=sortable&addIndex=0&hiddenColumns='))[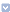](javascript:xmlhttpPost('/webqtl/main.py?FormID=AJAX_table',%20'sortable',%20'sort=human_chr&order=down&file=Mapping_CxSAkzH6&tableID=sortable&addIndex=0&hiddenColumns='))*** | *Mb Start (hg19)*  ***[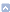](javascript:xmlhttpPost('/webqtl/main.py?FormID=AJAX_table',%20'sortable',%20'sort=mb_start_hg19&order=up&file=Mapping_CxSAkzH6&tableID=sortable&addIndex=0&hiddenColumns='))[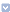](javascript:xmlhttpPost('/webqtl/main.py?FormID=AJAX_table',%20'sortable',%20'sort=mb_start_hg19&order=down&file=Mapping_CxSAkzH6&tableID=sortable&addIndex=0&hiddenColumns='))*** | *Gene Description*  ***[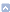](javascript:xmlhttpPost('/webqtl/main.py?FormID=AJAX_table',%20'sortable',%20'sort=description&order=up&file=Mapping_CxSAkzH6&tableID=sortable&addIndex=0&hiddenColumns='))[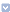](javascript:xmlhttpPost('/webqtl/main.py?FormID=AJAX_table',%20'sortable',%20'sort=description&order=down&file=Mapping_CxSAkzH6&tableID=sortable&addIndex=0&hiddenColumns='))*** | *PolymiRTS Database* [*>>*](http://compbio.uthsc.edu/miRSNP/) | *Gene Weaver Info Content* [*>>*](http://geneweaver.org/) |
| --- | --- | --- | --- | --- | --- | --- | --- | --- | --- | --- | --- |
| 1 | [Slit3](http://www.ncbi.nlm.nih.gov/entrez/query.fcgi?db=gene&cmd=Retrieve&dopt=Graphics&list_uids=20564) **[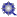](http://www.genenetwork.org/webqtl/main.py?cmd=sch&gene=Slit3&alias=1)** | [34.934957](http://genome.ucsc.edu/cgi-bin/hgTracks?clade=vertebrate&org=Mouse&db=mm9&position=chr11%3A34934957-35522005&pix=620&Submit=submit) | [587.048](javascript:centerIntervalMapOnRange2('11', 34.8762522, 35.5807098, document.changeViewForm)) | [647](http://www.genenetwork.org/webqtl/main.py?FormID=snpBrowser&chr=11&start=34.934957&end=35.522005&geneName=Slit3&s1=2&s2=3) | 1.102125 | -- | 5 | [168.025856](http://genome.ucsc.edu/cgi-bin/hgTracks?clade=vertebrate&org=Human&db=hg17&position=chr5:168025856-168660554) | slit homolog 3 (Drosophila... |  |  |
| 2 | [Pank3](http://www.ncbi.nlm.nih.gov/entrez/query.fcgi?db=gene&cmd=Retrieve&dopt=Graphics&list_uids=211347) **[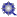](http://www.genenetwork.org/webqtl/main.py?cmd=sch&gene=Pank3&alias=1)** | [35.582996](http://genome.ucsc.edu/cgi-bin/hgTracks?clade=vertebrate&org=Mouse&db=mm9&position=chr11%3A35582996-35604787&pix=620&Submit=submit) | [21.791](javascript:centerIntervalMapOnRange2('11', 35.5808169, 35.6069661, document.changeViewForm)) | [2](http://www.genenetwork.org/webqtl/main.py?FormID=snpBrowser&chr=11&start=35.582996&end=35.604787&geneName=Pank3&s1=2&s2=3) | 0.091781 | -- | 5 | [167.915207](http://genome.ucsc.edu/cgi-bin/hgTracks?clade=vertebrate&org=Human&db=hg17&position=chr5:167915207-167939166) | pantothenate kinase 3 |  |  |
| 3 | [AI595406](http://www.ncbi.nlm.nih.gov/entrez/query.fcgi?db=gene&cmd=Retrieve&dopt=Graphics&list_uids=237730) **[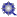](http://www.genenetwork.org/webqtl/main.py?cmd=sch&gene=AI595406&alias=1)** | [35.610881](http://genome.ucsc.edu/cgi-bin/hgTracks?clade=vertebrate&org=Mouse&db=mm9&position=chr11%3A35610881-35612386&pix=620&Submit=submit) | [1.505](javascript:centerIntervalMapOnRange2('11', 35.6107305, 35.6125365, document.changeViewForm)) | 0 | 0 | -- | -- | [--](http://www.genenetwork.org/webqtl/--) | expressed sequence AI59540... |  |  |
| 4 | [Rars](http://www.ncbi.nlm.nih.gov/entrez/query.fcgi?db=gene&cmd=Retrieve&dopt=Graphics&list_uids=104458) **[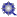](http://www.genenetwork.org/webqtl/main.py?cmd=sch&gene=Rars&alias=1)** | [35.621884](http://genome.ucsc.edu/cgi-bin/hgTracks?clade=vertebrate&org=Mouse&db=mm9&position=chr11%3A35621884-35648008&pix=620&Submit=submit) | [26.124](javascript:centerIntervalMapOnRange2('11', 35.6192716, 35.6506204, document.changeViewForm)) | [3](http://www.genenetwork.org/webqtl/main.py?FormID=snpBrowser&chr=11&start=35.621884&end=35.648008&geneName=Rars&s1=2&s2=3) | 0.114837 | -- | 5 | [167.846040](http://genome.ucsc.edu/cgi-bin/hgTracks?clade=vertebrate&org=Human&db=hg17&position=chr5:167846040-167878885) | arginyl-tRNA synthetase |  |  |
| 5 | [Wwc1](http://www.ncbi.nlm.nih.gov/entrez/query.fcgi?db=gene&cmd=Retrieve&dopt=Graphics&list_uids=211652) **[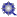](http://www.genenetwork.org/webqtl/main.py?cmd=sch&gene=Wwc1&alias=1)** | [35.652679](http://genome.ucsc.edu/cgi-bin/hgTracks?clade=vertebrate&org=Mouse&db=mm9&position=chr11%3A35652679-35793591&pix=620&Submit=submit) | [140.912](javascript:centerIntervalMapOnRange2('11', 35.6385878, 35.8076822, document.changeViewForm)) | [217](http://www.genenetwork.org/webqtl/main.py?FormID=snpBrowser&chr=11&start=35.652679&end=35.793591&geneName=Wwc1&s1=2&s2=3) | 1.539968 | -- | -- | [--](http://www.genenetwork.org/webqtl/--) | WW, C2 and coiled-coil dom... |  |  |
| 6 | [Odz2](http://www.ncbi.nlm.nih.gov/entrez/query.fcgi?db=gene&cmd=Retrieve&dopt=Graphics&list_uids=23964) **[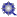](http://www.genenetwork.org/webqtl/main.py?cmd=sch&gene=Odz2&alias=1)** | [35.820170](http://genome.ucsc.edu/cgi-bin/hgTracks?clade=vertebrate&org=Mouse&db=mm9&position=chr11%3A35820170-36757745&pix=620&Submit=submit) | [937.575](javascript:centerIntervalMapOnRange2('11', 35.7264125, 36.8515025, document.changeViewForm)) | [3469](http://www.genenetwork.org/webqtl/main.py?FormID=snpBrowser&chr=11&start=35.82017&end=36.757745&geneName=Odz2&s1=2&s2=3) | 3.699971 | -- | -- | [--](http://www.genenetwork.org/webqtl/--) | odd Oz/ten-m homolog 2 (Dr... |  |  |
| 7 | [4930553C11Rik](http://www.ncbi.nlm.nih.gov/entrez/query.fcgi?db=gene&cmd=Retrieve&dopt=Graphics&list_uids=75315) **[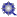](http://www.genenetwork.org/webqtl/main.py?cmd=sch&gene=4930553C11Rik&alias=1)** | [39.121204](http://genome.ucsc.edu/cgi-bin/hgTracks?clade=vertebrate&org=Mouse&db=mm9&position=chr11%3A39121204-39203727&pix=620&Submit=submit) | [82.523](javascript:centerIntervalMapOnRange2('11', 39.1129517, 39.2119793, document.changeViewForm)) | [73](http://www.genenetwork.org/webqtl/main.py?FormID=snpBrowser&chr=11&start=39.121204&end=39.203727&geneName=4930553C11Rik&s1=2&s2=3) | 0.884602 | -- | -- | [--](http://www.genenetwork.org/webqtl/--) | RIKEN cDNA 4930553C11 gene |  |  |

Table E. Candidate genes for Rat body weight between 162 and 172.5 MB on chr 2

| *Index*  ***[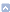](javascript:xmlhttpPost('/webqtl/main.py?FormID=AJAX_table',%20'sortable',%20'sort=index&order=up&file=Mapping_uiEOMv6f&tableID=sortable&addIndex=0&hiddenColumns='))[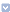](javascript:xmlhttpPost('/webqtl/main.py?FormID=AJAX_table',%20'sortable',%20'sort=index&order=down&file=Mapping_uiEOMv6f&tableID=sortable&addIndex=0&hiddenColumns='))*** | *Symbol*  ***[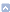](javascript:xmlhttpPost('/webqtl/main.py?FormID=AJAX_table',%20'sortable',%20'sort=symbol&order=up&file=Mapping_uiEOMv6f&tableID=sortable&addIndex=0&hiddenColumns='))[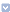](javascript:xmlhttpPost('/webqtl/main.py?FormID=AJAX_table',%20'sortable',%20'sort=symbol&order=down&file=Mapping_uiEOMv6f&tableID=sortable&addIndex=0&hiddenColumns='))*** | *Mb Start (rn3)*  ***[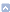](javascript:xmlhttpPost('/webqtl/main.py?FormID=AJAX_table',%20'sortable',%20'sort=mb_start_rn3&order=up&file=Mapping_uiEOMv6f&tableID=sortable&addIndex=0&hiddenColumns='))[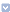](javascript:xmlhttpPost('/webqtl/main.py?FormID=AJAX_table',%20'sortable',%20'sort=mb_start_rn3&order=down&file=Mapping_uiEOMv6f&tableID=sortable&addIndex=0&hiddenColumns='))*** | *Length (Kb)*  ***[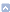](javascript:xmlhttpPost('/webqtl/main.py?FormID=AJAX_table',%20'sortable',%20'sort=length&order=up&file=Mapping_uiEOMv6f&tableID=sortable&addIndex=0&hiddenColumns='))[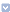](javascript:xmlhttpPost('/webqtl/main.py?FormID=AJAX_table',%20'sortable',%20'sort=length&order=down&file=Mapping_uiEOMv6f&tableID=sortable&addIndex=0&hiddenColumns='))*** | *Avg Expr* | *Mouse Chr*  ***[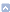](javascript:xmlhttpPost('/webqtl/main.py?FormID=AJAX_table',%20'sortable',%20'sort=mouse_chr&order=up&file=Mapping_uiEOMv6f&tableID=sortable&addIndex=0&hiddenColumns='))[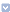](javascript:xmlhttpPost('/webqtl/main.py?FormID=AJAX_table',%20'sortable',%20'sort=mouse_chr&order=down&file=Mapping_uiEOMv6f&tableID=sortable&addIndex=0&hiddenColumns='))*** | *Mb Start (mm9)*  ***[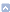](javascript:xmlhttpPost('/webqtl/main.py?FormID=AJAX_table',%20'sortable',%20'sort=mb_start_mm9&order=up&file=Mapping_uiEOMv6f&tableID=sortable&addIndex=0&hiddenColumns='))[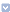](javascript:xmlhttpPost('/webqtl/main.py?FormID=AJAX_table',%20'sortable',%20'sort=mb_start_mm9&order=down&file=Mapping_uiEOMv6f&tableID=sortable&addIndex=0&hiddenColumns='))*** | *Human Chr*  ***[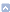](javascript:xmlhttpPost('/webqtl/main.py?FormID=AJAX_table',%20'sortable',%20'sort=human_chr&order=up&file=Mapping_uiEOMv6f&tableID=sortable&addIndex=0&hiddenColumns='))[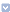](javascript:xmlhttpPost('/webqtl/main.py?FormID=AJAX_table',%20'sortable',%20'sort=human_chr&order=down&file=Mapping_uiEOMv6f&tableID=sortable&addIndex=0&hiddenColumns='))*** | *Mb Start (hg19)*  ***[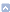](javascript:xmlhttpPost('/webqtl/main.py?FormID=AJAX_table',%20'sortable',%20'sort=mb_start_hg19&order=up&file=Mapping_uiEOMv6f&tableID=sortable&addIndex=0&hiddenColumns='))[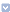](javascript:xmlhttpPost('/webqtl/main.py?FormID=AJAX_table',%20'sortable',%20'sort=mb_start_hg19&order=down&file=Mapping_uiEOMv6f&tableID=sortable&addIndex=0&hiddenColumns='))*** | *Gene Description*  ***[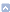](javascript:xmlhttpPost('/webqtl/main.py?FormID=AJAX_table',%20'sortable',%20'sort=description&order=up&file=Mapping_uiEOMv6f&tableID=sortable&addIndex=0&hiddenColumns='))[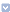](javascript:xmlhttpPost('/webqtl/main.py?FormID=AJAX_table',%20'sortable',%20'sort=description&order=down&file=Mapping_uiEOMv6f&tableID=sortable&addIndex=0&hiddenColumns='))*** |
| --- | --- | --- | --- | --- | --- | --- | --- | --- | --- |
| 1 | **[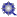](http://www.genenetwork.org/webqtl/main.py?cmd=sch&gene=Si&alias=1&species=rat)**[Si](http://www.ncbi.nlm.nih.gov/entrez/query.fcgi?db=gene&cmd=Retrieve&dopt=Graphics&list_uids=25588) | 163.471147 | [2.758](javascript:centerIntervalMapOnRange2('2', 163.470871, 163.474181, document.changeViewForm)) |  | 10 | 128.143313 | 3 | 166.179388 | Sucrase-isomaltase |
| 2 | **[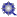](http://www.genenetwork.org/webqtl/main.py?cmd=sch&gene=Bche&alias=1&species=rat)**[**Bche**](http://www.ncbi.nlm.nih.gov/entrez/query.fcgi?db=gene&cmd=Retrieve&dopt=Graphics&list_uids=65036) | 164.279292 | [15.848](javascript:centerIntervalMapOnRange2('2', 164.277707, 164.296725, document.changeViewForm)) |  | 3 | 73.43973 | 3 | 166.973394 | butyrylcholinesterase |
| 3 | **[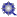](http://www.genenetwork.org/webqtl/main.py?cmd=sch&gene=Serpini2&alias=1&species=rat)**Serpini2 | 166.054562 | [20.579](javascript:centerIntervalMapOnRange2('2', 166.052504, 166.077199, document.changeViewForm)) |  | 3 | 75.046292 | 3 | 168.642424 | serine (or cysteine) proteinase inhibitor, clade I, member 2 |
| 4 | **[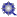](http://www.genenetwork.org/webqtl/main.py?cmd=sch&gene=MGC72992&alias=1&species=rat)**MGC72992 | 166.352897 | [0.324](javascript:centerIntervalMapOnRange2('2', 166.352865, 166.353253, document.changeViewForm)) |  |  |  |  |  | similar to programmed cell death 10 |
| 5 | **[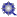](http://www.genenetwork.org/webqtl/main.py?cmd=sch&gene=Serpini1&alias=1&species=rat)**Serpini1 | 166.500871 | [2.907](javascript:centerIntervalMapOnRange2('2', 166.500580, 166.504069, document.changeViewForm)) |  | 3 | 75.361494 | 3 | 168.936224 | 166503778 |
| 6 | **[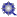](http://www.genenetwork.org/webqtl/main.py?cmd=sch&gene=BF394843&alias=1&species=rat)**BF394843 | 166.807619 | [0.227](javascript:centerIntervalMapOnRange2('2', 166.807596, 166.807869, document.changeViewForm)) |  |  |  |  |  | strongly similar to NP_780402.1 golgi phosphoprotein 4 |
| 7 | **[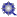](http://www.genenetwork.org/webqtl/main.py?cmd=sch&gene=Hspa8&alias=1&species=rat)**Hspa8 | 170.260397 | [0.539](javascript:centerIntervalMapOnRange2('2', 170.260343, 170.260990, document.changeViewForm)) |  | 9 | 40.609355 | 11 | 122.43341 | heat shock protein 8 |
| 8 | **[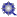](http://www.genenetwork.org/webqtl/main.py?cmd=sch&gene=BF407276&alias=1&species=rat)**BF407276 | 170.422566 | [0.421](javascript:centerIntervalMapOnRange2('2', 170.422524, 170.423029, document.changeViewForm)) |  |  |  |  |  | Rap guanine nucleotide exchange factor (GEF) 2 |
| 9 | **[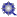](http://www.genenetwork.org/webqtl/main.py?cmd=sch&gene=AI410107&alias=1&species=rat)**AI410107 | 170.813123 | [0.550](javascript:centerIntervalMapOnRange2('2', 170.813068, 170.813728, document.changeViewForm)) |  |  |  |  |  |  |
| 10 | **[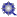](http://www.genenetwork.org/webqtl/main.py?cmd=sch&gene=RGD:1303174&alias=1&species=rat)**RGD:1303174 | 170.955118 | [2.899](javascript:centerIntervalMapOnRange2('2', 170.954828, 170.958307, document.changeViewForm)) |  |  |  |  |  | peptidylprolyl isomerase D |
| 11 | **[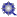](http://www.genenetwork.org/webqtl/main.py?cmd=sch&gene=RGD:735052&alias=1&species=rat)**RGD:735052 | 170.958922 | [1.079](javascript:centerIntervalMapOnRange2('2', 170.958814, 170.960109, document.changeViewForm)) |  |  |  |  |  | electron-transferring-flavoprotein dehydrogenase |
| 12 | **[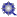](http://www.genenetwork.org/webqtl/main.py?cmd=sch&gene=RGD:735075&alias=1&species=rat)**RGD:735075 | 171.382526 | [0.362](javascript:centerIntervalMapOnRange2('2', 171.382490, 171.382924, document.changeViewForm)) |  |  |  |  |  | Unknown (protein for MGC:72614) |
| 13 | **[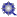](http://www.genenetwork.org/webqtl/main.py?cmd=sch&gene=Gria2&alias=1&species=rat)**Gria2 | 172.216279 | [0.556](javascript:centerIntervalMapOnRange2('2', 172.216223, 172.216891, document.changeViewForm)) |  | 3 | 80.488857 | 4 | 158.49952 | 172216835 |
| 14 | **[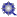](http://www.genenetwork.org/webqtl/main.py?cmd=sch&gene=Glrb&alias=1&species=rat)**Glrb | 172.401719 | [0.461](javascript:centerIntervalMapOnRange2('2', 172.401673, 172.402226, document.changeViewForm)) |  | 3 | 80.647525 | 4 | 158.354942 | glycine receptor, beta subunit |
